# Ice Converts Phenols into Persistent Mineral-Bound Polymers

**DOI:** 10.1021/acs.est.6c05553

**Published:** 2026-10-14

**Authors:** Yang Rong, Tao Luo, Tao Chen, Tra My Bui Thi, Khalil Hanna, Jean-François Boily

**Affiliations:** † Department of Chemistry, 105361 Umeå University, Umeå SE 901 87, Sweden; ‡ Université de Rennes, École Nationale Supérieure de Chimie de Rennes, CNRS, ISCR-UMR 6226, Rennes F-35000, France; § College of Chemistry, Chemical Engineering and Materials Science, Shandong Normal University, Jinan 250014, China

**Keywords:** freeze−thaw, manganese, polymerization, carbon stabilization, frozen soils

## Abstract

Freezing fundamentally alters how minerals transform dissolved phenols in cold environments. As water freezes, solutes are excluded from the crystal lattice and concentrated into microscopic pockets of unfrozen liquid, creating reaction conditions far more extreme than those of bulk solution. Here, we compare catechol oxidation on a key soil manganese oxide under freeze–thaw versus room-temperature conditions. Freezing redirects products from soluble humic-like substances to thermally stable, mineral-bound polymers. Freeze–thaw conditions removed over 50% of dissolved organic carbon within 24 h compared to less than 10% at room temperature, with solid-phase carbon increasing over 50-fold. The attenuation of the effect with increasing salinity is consistent with freeze-concentration as a key driver and indicates that the pathway should be strongest in freshwater and low-salinity frozen environments. Freeze–thaw conditions also converted preformed soluble products into mineral-bound forms, demonstrating that the process operates on a range of substrates. These findings reveal an unrecognized abiotic carbon stabilization pathway that could operate when microbial decomposition is suppressed by freezing with direct implications for carbon dynamics in permafrost and seasonally frozen environments.

## Graphical abstract

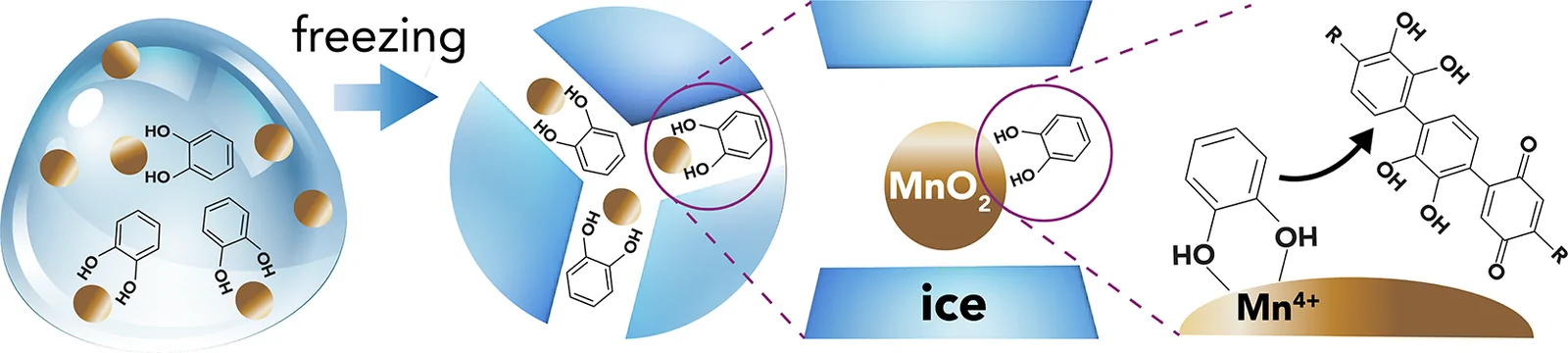

## 1 Introduction

Half of global soil carbon is stored in permafrost and seasonally frozen ground,^1–3^ yet the abiotic chemical processes that transform organic matter under frozen conditions are only beginning to be understood. As water freezes, solutes are excluded from the crystal lattice and concentrated into liquid intergrain boundaries (LIBs), microscopic pockets of unfrozen water within the ice matrix.^4^ Within these microenvironments, reactant concentrations increase dramatically, pH shifts, and redox potentials change, enabling reactions that do not occur in bulk solution.^5–7^ Notably, freeze-concentration can accelerate reactions, including conversion of plant-derived phenols into humic-like substances,^5^ aromatic oxidation,^6^ iron oxyhydroxide dissolution,^7,8^ and halide oxidation coupled to manganese dissolution.^9,10^

Abiotic humification of phenols concentrated at ice grain boundaries produces humic-like macromolecules through radical coupling, even without mineral catalysts.^5^ Separately, birnessite (MnO_2_) oxidizes halides in ice to generate reactive halogens that extensively functionalize natural organic matter,^9,10^ and freezing accelerates Mn­(III) generation at Mn­(IV) oxide surfaces. Under ambient conditions, birnessite can readily oxidize and polymerize phenolic dissolved organic matter.^11^ Yet, no study has combined reactive minerals with phenolic substrates under freezing conditions to determine whether mineral-catalyzed polymerization and carbon stabilization occur in ice.

Understanding this pathway matters for predicting carbon fate as permafrost is increasingly thawed and refrozen under climate change. The “mineral carbon pump” concept highlights how soil minerals stabilize organic carbon against microbial degradation, with iron and manganese oxides accounting for 20–40% of carbon stabilization in soils.^3,12–14^ Whether freezing strengthens or disrupts these mineral-organic associations will influence how much carbon is released as permafrost thaws, yet most studies have examined bulk carbon fluxes rather than the underlying molecular mechanisms.^1,3,15,16^

Birnessite provides an ideal system for addressing this question. This layered manganese oxide is among the most abundant in soils^20^ and occurs across environments from temperate forests to permafrost active layers,^21^ and its Mn­(IV) centers make it highly effective at oxidizing polyphenols (Figure 1).^22,23^ Recent work further showed that freezing induces Mn vacancies in birnessite and enhances its oxidative reactivity toward bisphenol.^24^ Catechol, a polyphenol released during lignin degradation and a key precursor to humic substances,^25^ undergoes well-characterized oxidation on birnessite. Under ambient conditions, this reaction produces soluble oligomeric products. Polyphenols constitute 1 to 5% of soil organic carbon in boreal ecosystems,^26,27^ and their fate during freeze–thaw cycling, whether retained as dissolved humic substances or converted to mineral-bound forms, has direct implications for carbon export and long-term storage.^28,29^

**Figure 1:**
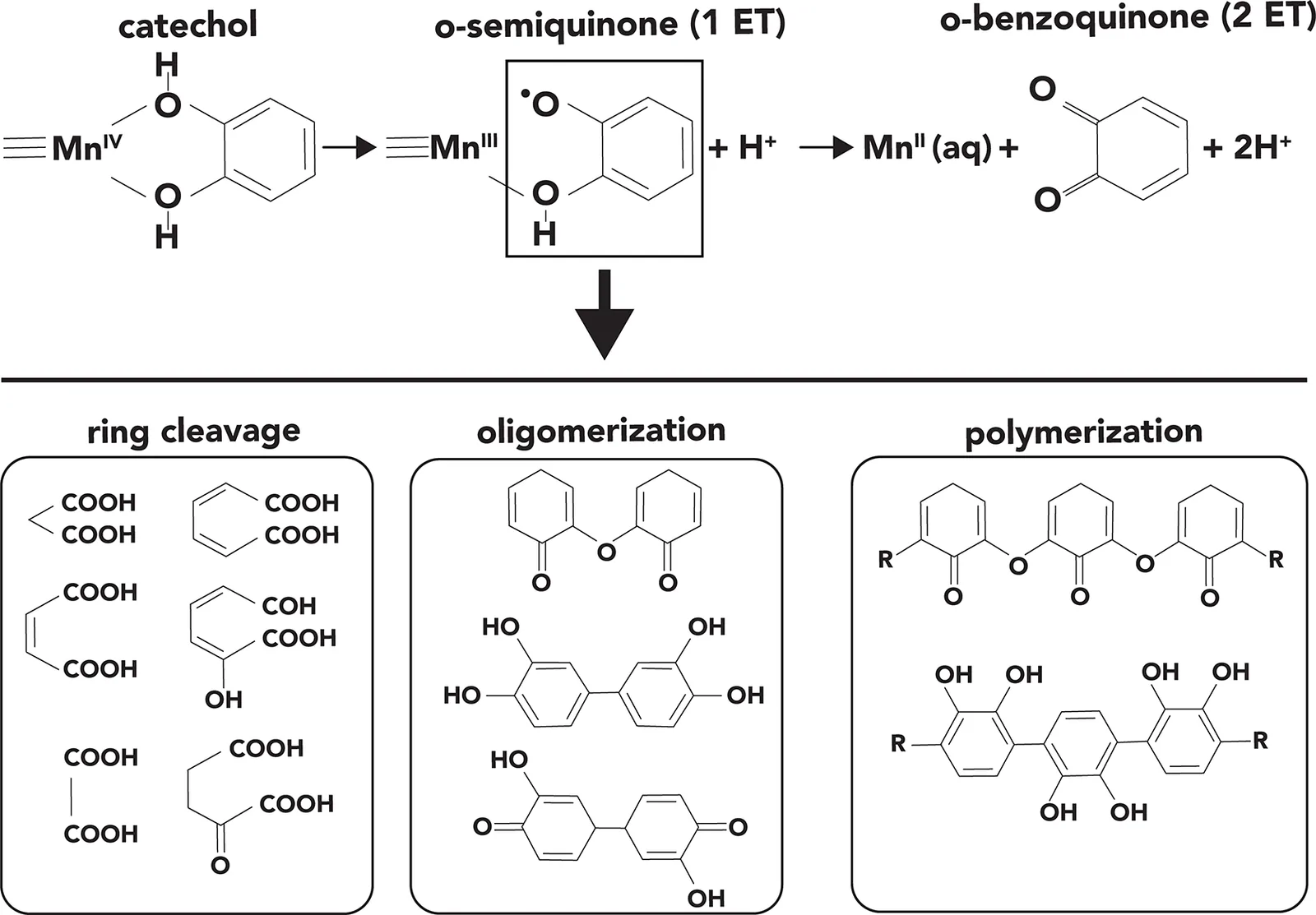
Catechol–birnessite reaction pathways. Surface coordination to Mn­(IV) sites initiates electron transfer, producing *o*-semiquinone (1 ET, one-electron transfer) and *o*-benzoquinone (2 ET, two-electron transfer) with concurrent Mn reduction. Reactive intermediates can undergo ring cleavage or oxidative coupling to form oligomeric and polymeric products, as summarized from previous studies.^17–19^

The central question is whether LIB conditions shift catechol-birnessite products from soluble oligomers toward insoluble polymers with enhanced thermal stability. Such a shift would represent an unrecognized abiotic carbon stabilization pathway operating when microbial activity is suppressed by freezing, potentially extending to the broader dissolved organic carbon pool. Here, we demonstrate that freeze–thaw cycles (FTCs) dramatically redirect product distribution toward mineral-bound polymers, directly imaging active chemistry within frozen microenvironments and constraining the mechanism’s environmental boundaries through salinity and pH dependence.

## 2 Materials and Methods

### 2.1 Mineral Synthesis

Acid birnessite was synthesized by reacting KMnO_4_ (Sigma-Aldrich) and HCl in boiling water.^30^ The resulting suspension was purged of dissolved atmospheric CO_2_(g) using a stream of N_2_(g) and stored in the dark at 4 °C.

### 2.2 Confocal Raman Microscopy

Raman measurements of catechol-bearing birnessite suspensions (500 μM catechol, 50 mg L^–1^ birnessite, 100 mM NaCl, pH 7) and of catechol solutions (500 μM catechol in 100 mM NaCl at pH 7) were performed using a Renishaw inVia Qontor confocal Raman microscope equipped with a 532 nm laser and a Leica 50× long working distance objective. A 10 μL solution or suspension droplet was deposited on a Linkam THMS600 stage for freezing and cooled from 25 to −20 °C at a rate of 10 °C min^–1^. Samples were maintained at −20 °C, and Raman spectra were collected 30 min after the onset of cooling without intervening thawing.

### 2.3 Freeze–Thaw Experiments

Stock solutions of catechol (p*K*_a_,_1_ = 9.36, p*K*_a_,_2_ = 12.98) were freshly prepared before experiments. Under all test conditions, catechol was chiefly present in its neutral, uncharged form (C_6_H_4_(OH)_2_). Batch experiments were performed in clear 15 mL polyethylene tubes containing 50 μM catechol and 50 mg L^–1^ birnessite in 1, 30, or 100 mM NaCl. Experiments for TOC analyses were performed using 10-fold higher concentrations (500 μM catechol and 500 mg L^–1^ birnessite), as the standard catechol concentration (50 μM) was near the instrument detection limit. Those for manganese redox-state analyses were performed using 500 μM catechol and 50 mg L^‑1^ birnessite. The pH was adjusted to 5, 7, or 9 using small aliquots of 1 M HCl or NaOH. Each 60 min FTC comprised freezing at −20 °C (20 min), warming to 20 °C (10 min), holding (20 min), and cooling to −20 °C (10 min) using a programmed ethanol bath (Julabo 449F). Controls were maintained at 20 °C. The 60 min cycle was repeated continuously until each sampling point such that the cumulative reaction time corresponded directly to the number of completed FTCs (e.g., 2 h = 2 FTCs and 24 h = 24 FTCs). The pH of RT and FTC samples was measured at 20 °C, with FTC samples completely thawed before measurement. All reactions were conducted in the dark without oxygen removal. Samples were collected at 0, 1, 2, 4, 6, 24, and 48 h.

Supernatants of the reacted samples were collected by centrifugation (4,200 rpm, 20 min) followed by filtration (0.45 μm). Solution-phase products were characterized by (i) UV–vis spectrophotometry (Cary-50, Varian, 200–700 nm), (ii) high-performance liquid chromatography (HPLC; UV detection at 275 nm; Waters 600, C18 column), (iii) liquid-chromatography–mass-spectrometry (Agilent 6230 TOF), and (iv) fluorescence excitation–emission matrix spectroscopy (Xenon source, PerkinElmer) with parallel factor analysis (PARAFAC) modeling.^31^ See Supporting Information for details on methods.

Solids collected by centrifugation were dried under a flow of N_2_(g) and analyzed by (i) X-ray photoelectron spectroscopy (XPS; Kratos Axis Ultra, monochromatic Al Kα) for Mn oxidation state determination^32^ and carbon speciation, (ii) Fourier transform infrared spectroscopy (Bruker Vertex 70/V), (iii) powder X-ray diffraction (XRD; Bruker D8 Advance), (iv) transmission electron microscopy (JEOL JEM-2100), (v) thermogravimetric analysis (TGA) coupled to real-time CO_2_ quantification by nondispersive infrared analysis (TA Instruments TGA 5500/LI-COR LI-7000), and (vi) elemental carbon analysis by combustion (Elementar UNICUBE, 670 °C). See Supporting Information for details on methods.

Finally, CO_2_(g) was quantified by nondispersive infrared analysis. A 22.5 mL min^–1^ stream of *N*_2_(*g*) was injected directly through the suspension via a needle inserted through an airtight septum, stripping dissolved and headspace CO_2_. The outlet gas stream was directed to the detector, and CO_2_(g) evolution was monitored continuously over a 75 min period.

### 2.4 Carbon Analysis

The organic matter content was quantified using a TOC-V CSH analyzer (Shimadzu). Three measurement approaches were used: (i) soluble organic carbon was determined by acidifying filtrates to pH 2 to remove inorganic carbon and minimize atmospheric CO_2_ incorporation; (ii) organic matter adsorption on birnessite was assessed by reductively dissolving birnessite with excess hydroxylamine hydrochloride, followed by centrifugation and supernatant TOC analysis (pristine birnessite controls confirmed complete dissolution); and (iii) carbon mass balance across liquid and solid phases was verified by dissolving birnessite without prior centrifugation or filtration. Dissolution was performed with magnetic stirring to ensure aeration and even mixing during sampling.

Carbon content was determined by combustion at 670 °C using an Elementar UNICUBE elemental analyzer with thermal conductivity detection (TCD) of CO_2_. Values were obtained from a standard curve and expressed as weight percent of the total sample mass, which here includes structural water in birnessite.

### 2.5 Extraction of Catechol-Derived OC Associated with Birnessite

The desorption stability of organic carbon (OC) in birnessite-catechol reaction solids was assessed by aqueous extraction under circumneutral, acidic, and alkaline conditions.^33–36^ Wet reaction slurries containing 5 g L^–1^ dry solid were prepared in ultrapure water, acidified ultrapure water (pH 3.5, with a few μL of concentrated HCl), or 0.1 M NaOH (pH 13), and agitated end-over-end for 24 h at room temperature. Solids were recovered by centrifugation and dried under N_2_(g) for elemental C analysis. All treatments were performed in triplicate.

NaOCl oxidation was used as an operational chemical proxy for oxidative degradation, distinguishing NaOCl-removable from NaOCl-resistant OC without directly assessing susceptibility to microbial or enzymatic degradation.^33–35^ To evaluate the susceptibility of mineral-associated OC to NaOCl oxidation, the wet reaction slurry was mixed with NaOCl (1 M initial concentration, adjusted to pH 3.5 with concentrated HCl to minimize pH-driven desorption) at 10 g dry solid L^–1^ and agitated end-over-end at room temperature for 6 h.^36^ Solids were recovered by centrifugation and dried under N_2_(g) for elemental C analysis in triplicate. OC remaining after NaOCl treatment was defined as the NaOCl-resistant fraction, and OC removed during treatment as the NaOCl-removable fraction.

Given the strong pH dependence of organic functional group interactions with metal oxides, ultrapure water adjusted to pH 7 under N_2_(g) before extraction represents the least aggressive condition for OC release, whereas alkaline extraction (pH 12) and NaOCl oxidation promote the greatest OC removal through base-driven desorption/dissolution and chemical oxidation, respectively.

## 3 Results

We first examined the spatial distribution of catechol and birnessite within frozen samples using confocal Raman microscopy. Both reactants colocalized within liquid intergrain boundaries (LIBs) (Figure 2a), while ice crystal domains remained solute-depleted. Raman spectra collected 30 min after the onset of cooling, while the samples remained frozen at −20 °C, showed characteristic changes in catechol chemistry within these concentrated microenvironments (Figure 2b,c). The relative intensity of C–H bands decreased, while a band at 1474 cm^–1^ became predominant. This band may include contributions from phenoxyl-like intermediates formed during catechol oxidation.^37^ The increased prominence of the phenoxy-associated band is consistent with enhanced formation of phenoxyl-like intermediates during catechol transformation.^38^ The relative attenuation of C–H bands is consistent with aromatic substitution during oligomerization or polymerization (Figure S1). These spectral changes thereby confirm that catechol underwent active chemical transformation within LIBs during freezing, rather than simply being preserved.

**Figure 2:**
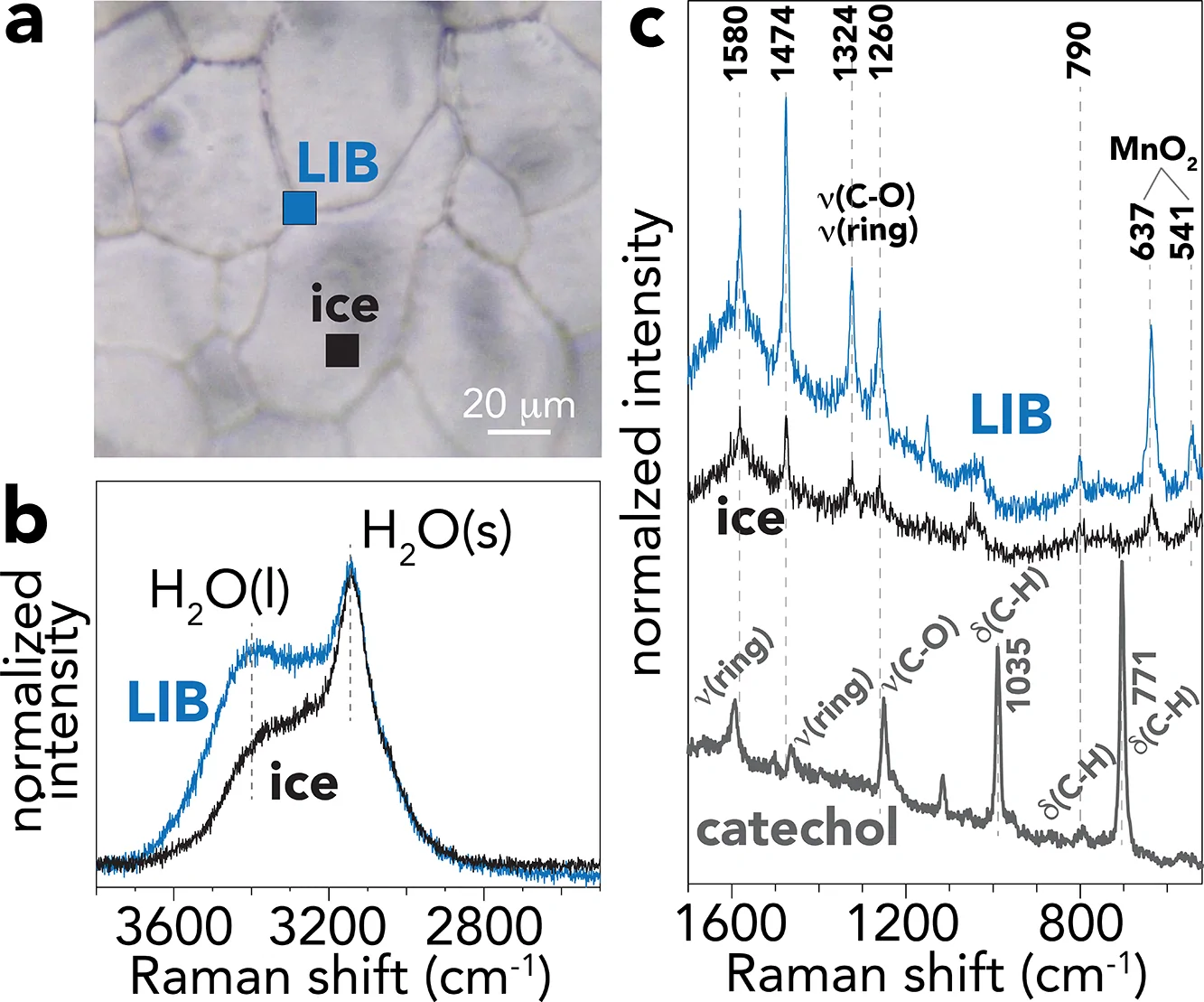
Direct visualization of LIB microenvironments by confocal Raman microscopy. (a) Optical image of frozen catechol–birnessite suspension showing ice crystal domains and LIB regions. Scale bar, 20 μm. (b) Raman spectra of the water stretching region (3600 to 2800 cm^–1^) comparing liquid-like LIB signatures versus ice crystal regions. Intensity was normalized for the band of I_h_ H_2_O(s). (c) Raman spectra of the organic fingerprint region (400–1600 cm^–1^) collected from LIB and ice-crystal regions. Bands at 1580 cm^–1^ (C=C), 1474 cm^–1^ (possible phenoxy species), 1324 cm^–1^ (C–O), and 1260 cm^–1^ (C–O) reveal differences in the organic species present in the two microenvironments. Bands at 637 and 541 cm^–1^ in the LIB spectrum correspond to birnessite Mn–O vibrations, confirming mineral colocalization with organic species. Spectra were baseline-corrected by subtracting the minimum intensity near 950 cm^–1^ and subsequently normalized to the intensity at the low-wavenumber end point. Relative band intensities were then compared at identical Raman shifts. Reference spectrum of catechol shown for comparison. Conditions: 500 μM catechol, 50 mg L^–1^ birnessite, 100 mM NaCl, −20 °C, pH_0_ = 7.0.

Having established that chemical transformation occurs within frozen microenvironments, we next quantified its consequences. Samples were exposed to freeze–thaw cycles (FTCs) spanning 1 to 48 h. Each 60 min FTC consisted of freezing at −20 °C, warming to 20 °C, holding, and then cooling back to −20 °C. The 60 min cycle was repeated continuously such that each sampling time corresponded directly to the cumulative number of completed FTCs (e.g., 2 h = 2 FTCs). Control experiments without birnessite confirmed that mineral-mediated redox chemistry was required for substantial polymerization (Figure S2), while experiments at 20 °C (RT) provided baseline comparisons.

### 3.1 Carbon Fate

Freezing produced a striking divergence in carbon partitioning between dissolved and solid fractions. Total organic carbon (TOC) analysis revealed fundamentally different reaction trajectories between RT and FTC conditions (Figure 3a). FTC at pH 7 in 1 mM NaCl removed ∼53% of dissolved carbon within 24 h (38.5 to 18 mg L^–1^) versus only ∼8% at RT (37.1 to 34 mg L^–1^), with complete mass balance confirmed between dissolved and solid fractions.

**Figure 3:**
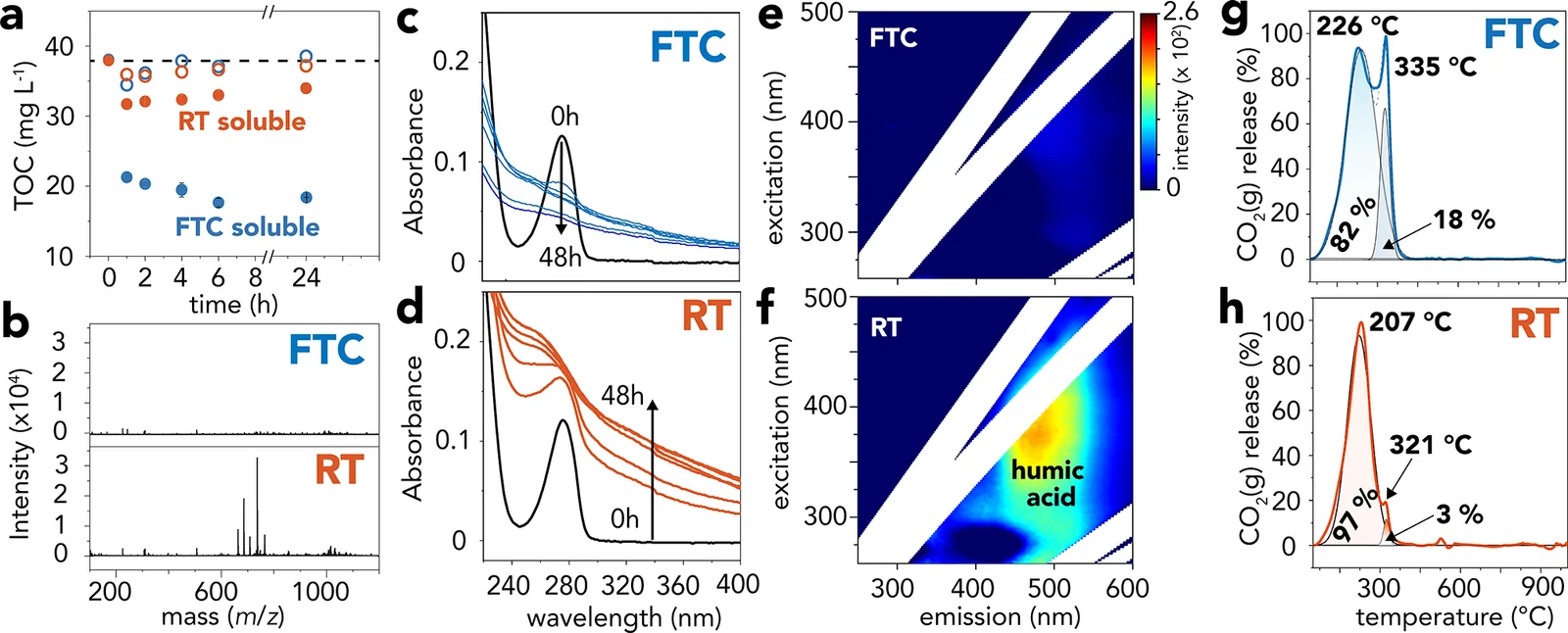
Carbon fate under FTC versus RT conditions. (a) TOC evolution showing divergent carbon partitioning. Open symbols represent total organic carbon across solid and liquid phases (dashed line as guide). Filled symbols represent soluble fractions. Error bars represent the standard deviation (mean ± SD) of triplicate measurements. (b) LC-MS spectra after 48 h. FTC (top) shows minimal soluble products, whereas RT (bottom) yields abundant species in the *m*/*z* 600–800 range. (c, d) Time-resolved UV–vis spectra (0–48 h) under (c) FTC and (d) RT. FTC shows progressive absorbance decline with near-complete disappearance of the catechol peak (275 nm) within 1 h (black line, *t* = 0 h), whereas RT shows increasing absorbance consistent with accumulating soluble aromatic products. (e, f) Fluorescence excitation–emission (Ex–Em) matrices after 48 h under (e) FTC and (f) RT. FTC shows negligible fluorescence, whereas RT shows intense humic-like signatures (Ex/Em ≈ 375/475 nm). White regions indicate masked Rayleigh and Raman scattering. (g, h) Temperature-programmed CO_2_ evolution from (g) FTC and (h) RT solids. FTC shows peaks at 226 °C (82%) and 335 °C (18%), RT at 207 °C (97%) and 321 °C (3%). Shaded areas denote relative contributions of each thermal population, obtained by Gaussian deconvolution. Conditions: [catechol]_0_ = 50 μM, [MnO_2_] = 50 mg L^–1^, pH_0_ = 7.0, [NaCl] = 1 mM (b–f); [catechol]_0_ = 500 μM, [MnO_2_] = 500 mg L^–1^, pH_0_ = 7.0, [NaCl] = 1 mM (a, g, h).

Combustion analysis (Figure S3) showed that FTC solids contained ∼52 times more carbon (2.6% w/w) than RT (0.05% w/w). Liquid chromatography–mass spectrometry (LC-MS) corroborated this divergence (Figure 3b) by showing that RT produced abundant soluble compounds with *m*/*z* values in the 600–800 range, while FTC samples were nearly indistinguishable from blanks.^39^ Reductive dissolution experiments (Figure S4) released no additional carbon to solution, while separate CO_2_ monitoring detected no CO_2_ production (Figure S5). This ruled out enhanced adsorption and oxidative mineralization as explanations for the divergent partitioning.

The two pathways also diverged in the optical signatures of the samples. Under FTC, UV–visible spectra of the filtered solutions (Figure 3c) revealed rapid catechol depletion within the first hour, with overall absorbance declining progressively, indicating that reaction products were removed from solution rather than accumulating. Under RT (Figure 3d), catechol persisted longer, while absorbance increased steadily, consistent with accumulating soluble aromatic products. Both conditions generated a new absorption band (π→π* transition at ∼250 nm) characteristic of newly formed conjugated aromatic structures. Fluorescence spectroscopy (Figure 3e,f; Figures S6–S11) showed that RT products had characteristic humic-like signatures,^40–42^ while FTC exhibited minimal fluorescence, consistent with carbon transfer to the solid phase.

Temperature-programmed CO_2_ evolution and chemical extraction tests further characterized the FTC and RT solids. Temperature-programmed experiments revealed greater thermal stability in FTC solids (Figure 3g). The main CO_2_ evolution peak shifted upward by 19 °C (207 to 226 °C), and the proportion of the high-temperature component was six times greater in FTC (18%) than in RT (3%) solids, with the peak shifting from 321 to 335 °C (Figure 3h). TGA confirmed this shift toward thermally resistant forms (Figure S12). Chemical stability tests showed that FTC-derived carbon was largely retained after acid extraction (81% at pH 3.5), alkaline extraction (73% at pH 13), and NaOCl oxidation (54–67% retained) (Figure S13). Taken together, the carbon partitioning, spectroscopic, and stability data establish that FTC and RT produce fundamentally different products from the same reactants.

### 3.2 Coupled Carbon Incorporation and Manganese Reduction

The solid-phase differences between FTC and RT extend beyond carbon to the minerals themselves. XPS (Figure S14) revealed that carbon enrichment and manganese reduction proceeded in tandem. Surface carbon loading increased from C/Mn = 0.3 (unreacted birnessite) to 0.62 (RT) to 1.22 (FTC), with the largest increase occurring in the C–C/C–H component under FTC (Figure 4a; Table S3). The Mn-normalized O 1s components assigned to a combined C=O/Mn–OH component and a separate C–OH component were likewise enriched under FTC (Figure 4b; Table S3), consistent with cross-linked catechol-derived polymers incorporating oxygen functionalities. Vibrational spectroscopy showed intense aromatic C=C and C–O signatures in FTC solids compared to weak signals in RT products (Figure S15).^25^

**Figure 4:**
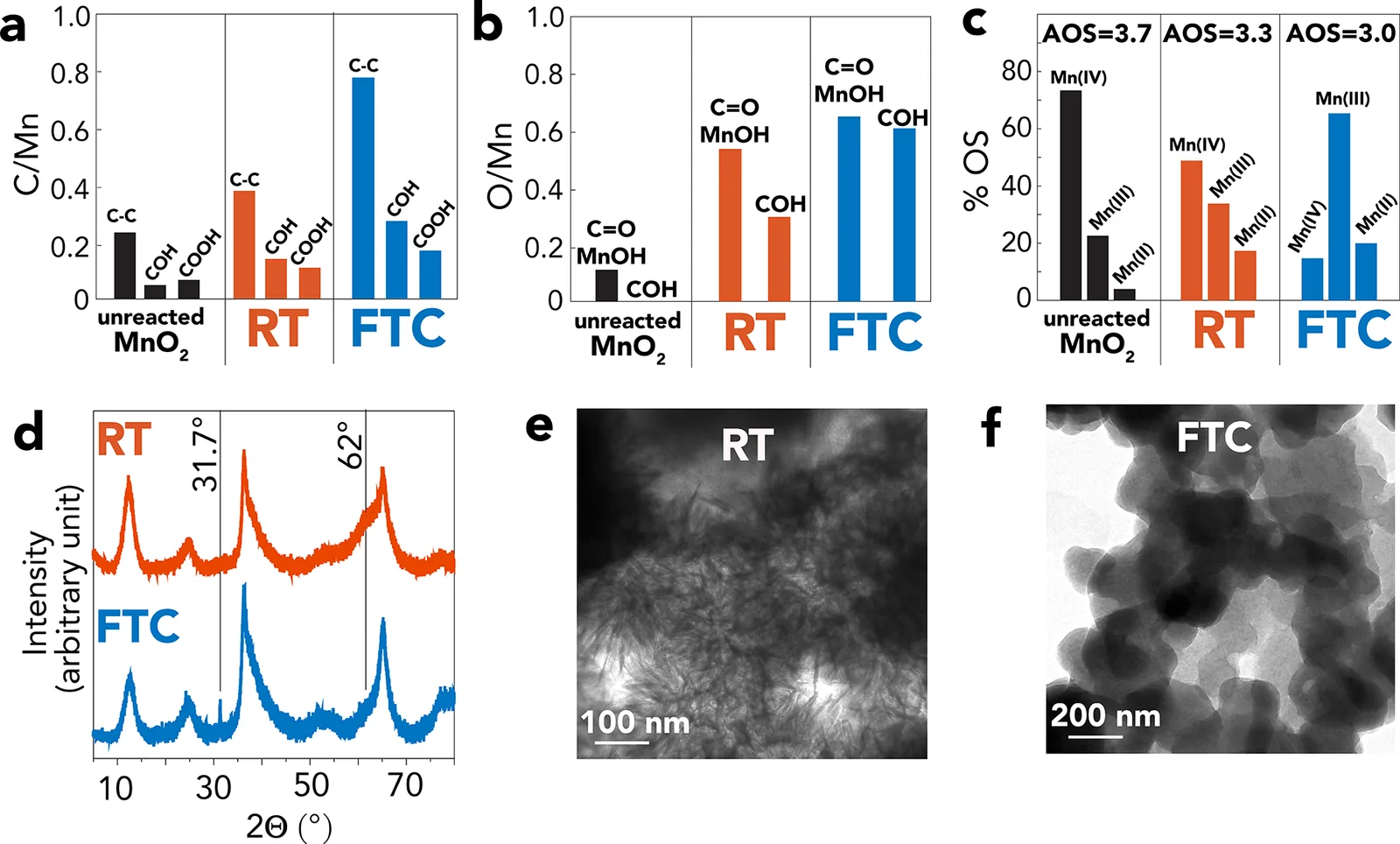
Solid-phase characterization under FTC and RT conditions. (a) XPS-derived carbon species normalized to Mn (C/Mn) for unreacted birnessite, RT, and FTC solids, showing C–C/C-H, C–OH, and COOH components. (b) XPS-derived oxygen species normalized to Mn (O/Mn) showing a combined C=O/MnOH, and a separate COH components. (c) Mn oxidation state distribution (% OS) for unreacted birnessite (average oxidation state, AOS = 3.7), RT (AOS = 3.3), and FTC (AOS = 3.0). (d) Powder XRD patterns for RT (red) and FTC (blue). Both retain dominant birnessite reflections (∼12° and ∼25° 2θ). FTC shows a new sharp reflection at 31.7° 2θ. The latter is not assigned to a specific Mn-bearing phase because additional diagnostic reflections are absent. (e) TEM of RT solid showing layered birnessite morphology. Scale bar, 100 nm. (f) TEM of FTC solid showing spherical nanocrystalline particles with distinct grain boundaries. Scale bar, 200 nm. Conditions: [catechol]_0_ = 500 μM, [MnO_2_] = 500 mg L^–1^, pH_0_ = 7.0, [NaCl] = 1 mM, reaction time = 2 days (a, b); [catechol]_0_ = 500 μM, [MnO_2_] = 50 mg L^–1^, pH_0_ = 7.0, [NaCl] = 1 mM, reaction time = 2 days (c–f).

The average manganese oxidation state decreased correspondingly from 3.7 (unreacted birnessite) to 3.3 (RT) to 3.0 (FTC) (Figure 4c; Figure S16; Table S4), and vibrational spectroscopy showed progressive shifts in Mn–O stretching frequencies consistent with phases of lower Mn oxidation state (Figure S17). X-ray diffraction (XRD) of the FTC solid (Figure 4d) showed that the dominant birnessite reflections (∼12° and ∼25° 2θ) persisted. A sharp reflection appeared at 31.7° 2θ, but the absence of additional diagnostic reflections precluded assignment to a specific Mn-bearing phase. Its position is also consistent with crystalline NaCl. Transmission electron microscopy (TEM) nevertheless showed a clear morphological transformation. Here, RT solids retained the layered birnessite morphology (Figure 4e), whereas FTC solids appeared as spherical nanocrystalline particles with distinct grain boundaries (Figure 4f). The C/Mn ratios and Mn oxidation states thus covaried systematically across unreacted birnessite, RT, and FTC conditions, linking the degree of carbon incorporation directly to the extent of manganese reduction.

### 3.3 Environmental and Substrate Controls on Freeze-Concentration Reactivity

Multiple lines of evidence support freeze concentration as a major driver of the observed product shift (Figure 5). Temperature-resolved kinetics rule out a purely thermal explanation, while the salinity dependence provides direct evidence that the effect scales with the degree of freeze concentration. Post-thaw pH trajectories (Figure 5f; Figure S18) provide an additional constraint on reaction chemistry, and experiments with preformed soluble products show that the mechanism extends beyond fresh monomers.

**Figure 5:**
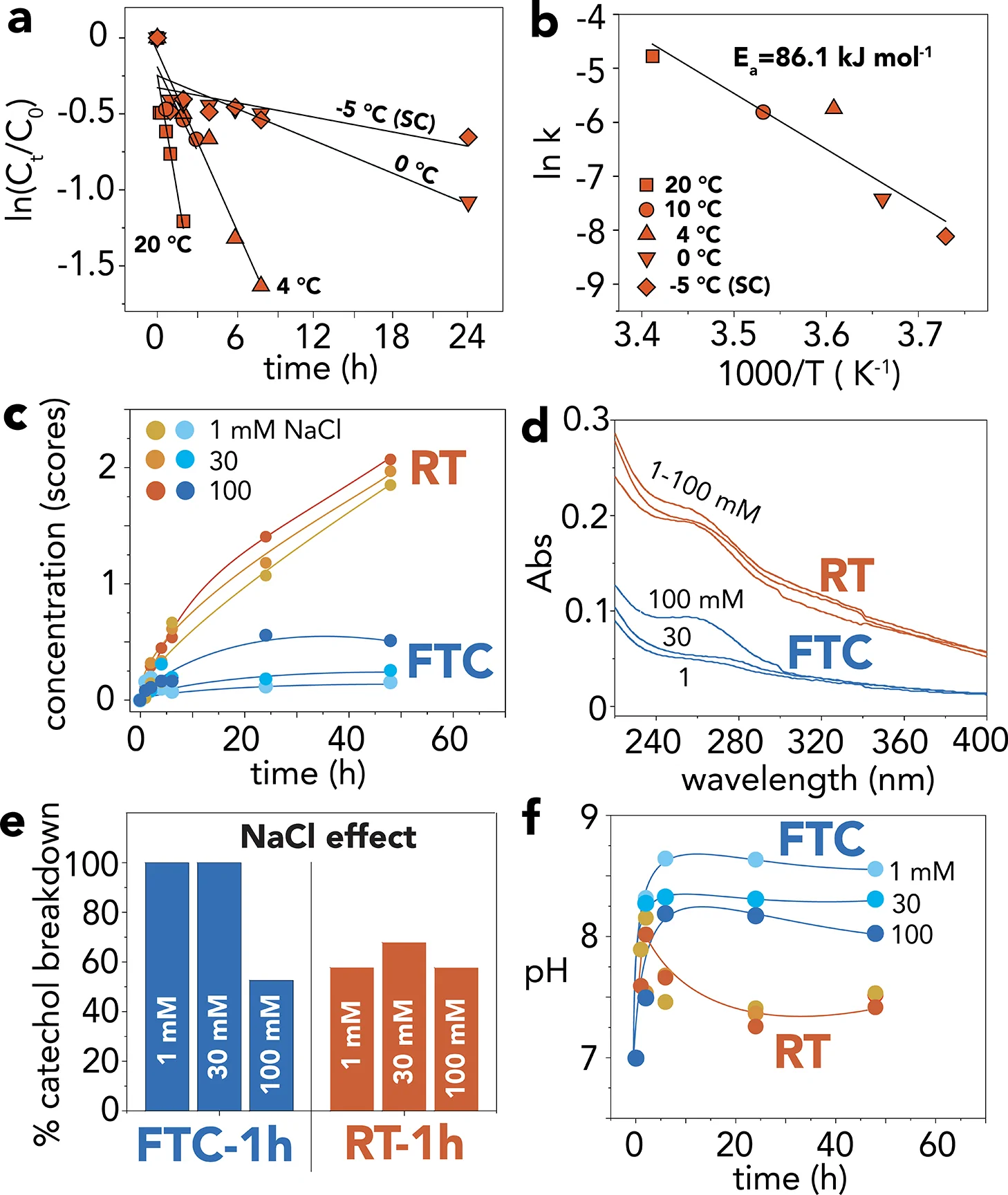
Environmental controls on freeze-concentration reactivity. (a) Pseudo-first-order plots of parent catechol depletion, ln­(C/C_0_), measured by HPLC in liquid or supercooled water at 20, 10, 4, 0, and −5 °C (cf. legend in b; SC=supercooled). Lines are linear fits used to obtain the apparent first-order rate constants. (b) Arrhenius plot of the apparent first-order rate constant, yielding an apparent activation energy of 86.1 kJ mol^–1^. Line is a linear fit. (c) PARAFAC scores tracking soluble humic-like substance accumulation over 48 h at 1, 30, and 100 mM NaCl. RT (red) shows steady accumulation that increases with ionic strength, whereas FTC (blue) shows suppressed accumulation at all salinities, with progressive attenuation of the FTC effect as ionic strength increases. Lines connect data points for visual clarity and do not represent fitted functions. (d) UV–vis spectra after 2 days. RT spectra (red) overlap across all salinities (1–100 mM), whereas FTC spectra (blue) show progressively increasing residual absorbance with salt concentration, approaching RT behavior at 100 mM. (e) Catechol breakdown after 1 h. FTC achieves near-complete breakdown at 1 and 30 mM NaCl but drops to ∼50% at 100 mM, converging with RT. (f) pH evolution under FTC (blue) and RT (red) at 1, 30, and 100 mM NaCl. FTC conditions maintain higher post-thaw pH values of approximately 8.0–8.5 regardless of salinity, while RT shows an initial increase followed by gradual acidification. Lines connect data points for visual clarity and do not represent fitted functions. Conditions: [catechol]_0_ = 50 μM, [MnO_2_] = 50 mg L^–1^, pH_0_ = 7.0, [NaCl] = 1 mM (a, b), 1–100 mM (c–f).

Catechol depletion rates measured in liquid and supercooled water at an initial pH of 7.0 generally decreased with decreasing temperature, yielding an apparent activation energy of 86.1 kJ mol^–1^ (Figure 5a,b). Thus, the enhanced reactivity observed during FTC at −20 °C cannot be attributed to cooling alone. Because reaction kinetics are also pH dependent, the temperature series does not by itself distinguish all contributions of freezing from changes in reaction chemistry. However, the systematic attenuation of the FTC effect with increasing initial NaCl concentration independently supports freeze-concentration and confinement as important drivers of the enhanced reaction.

Salinity experiments provided a direct test of this interpretation. During freezing, NaCl is excluded from the ice lattice and concentrated in the remaining LIB. According to the equilibrium NaCl–H_2_O phase relationship, cooling to −20 °C, just above the eutectic temperature (−21.1 °C), drives the residual liquid toward a NaCl concentration of ∼4.5 M, largely independent of the initial NaCl concentration. The initial salinity instead controls the amount of liquid water retained, as lower initial NaCl concentrations produce smaller LIB fractions at a given temperature.

This difference in LIB volume strongly affects the freeze-concentration of species whose initial concentrations are held constant. Because catechol was held constant across the salinity series, progressively lower initial NaCl concentrations confine the same amount of catechol into progressively smaller liquid volumes, thereby increasing its nominal concentration within the LIB. Birnessite particles are likewise excluded from growing ice crystals and concentrated within interstitial regions, increasing contact between the mineral and freeze-concentrated catechol. Thus, increasing the initial NaCl concentration increases the amount of LIB water and reduces the concentration factor experienced by catechol and other non-NaCl components, consistent with the observed attenuation of the FTC effect.^43,44^ Fluorescence analysis^31^ at 1, 30, and 100 mM NaCl (Figure 5c; Figures S19–S22) supported this picture. Under FTC, humic-like products were suppressed at low salinity but accumulated progressively with increasing NaCl, approaching RT behavior at 100 mM. UV–vis spectra after 2 days showed the same convergence (Figure 5d). Catechol breakdown remained complete under FTC at 1 and 30 mM NaCl but decreased to ∼50% at 100 mM (Figure 5e), mirroring incomplete breakdown under RT at the same salinity. Together, these results (Figure S23) support a central role for LIB volume, and the resulting freeze-concentration of catechol and mineral particles, in controlling the partitioning between soluble and mineral-associated products.

pH trajectories provided an additional constraint (Figure 5f). For experiments initiated at pH 7.0, FTC samples consistently showed higher post-thaw pH values, reaching about 8.0–8.5 across all NaCl concentrations tested. Under RT conditions, pH initially increased, consistent with a net proton-consuming stage that may include reductive dissolution of Mn­(IV), before gradually declining as the reaction progressed. Under FTC, the post-thaw pH remained higher, consistent with a different net proton balance associated with the greater extent of Mn reduction and altered reaction pathways. Because these measurements were made only after complete thawing at 20 °C, they do not establish the *in situ* pH or proton balance within the LIBs. Similar differences between FTC and RT product distributions were also observed in experiments initiated at pH 5 and 9 (Figures S24–S29), showing that the FTC effect is not restricted to an initial pH of 7.0. Previous studies^17,18^ have nevertheless shown that pH can regulate Mn oxide-mediated phenolic transformation, with acidic conditions favoring oxidative decomposition and aromatic ring cleavage, whereas circumneutral to mildly alkaline conditions favor condensation and polymerization.

Finally, the mechanism is not limited to the fresh monomeric substrates. Under FTC, UV–vis absorbance, TOC, and fluorescence intensity continued declining beyond the first hour (Figure 3a,c,e) even after catechol was largely consumed. The supernatant from RT reactions after 2 days, containing accumulated soluble polymers, was mixed with fresh birnessite and subjected to a single FTC. UV–visible absorbance decreased immediately (Figure S30), while controls without birnessite showed no change, confirming that mineral-mediated chemistry is required for this secondary processing. The mechanism thus extends to soluble humic-like substances at various stages of transformation, broadening its relevance from a single model compound to the natural dissolved organic carbon pool.

## 4 Discussion

Freeze concentration within LIBs transforms a diffusion-limited reaction into a high-frequency collision environment that kinetically favors radical coupling and polymer growth over the soluble oligomers produced under ambient conditions. The emergence of a phenoxy-associated Raman band (Figure 2c) is consistent with radical-mediated catechol transformation within these confined spaces, where elevated reactant concentrations drive the pathway toward solid-phase products.

The carbon partitioning data establish that freezing controls product fate at the molecular level. The controls against simple adsorption and oxidative mineralization indicate that FTC transfers carbon into a strongly mineral-associated, poorly extractable solid fraction. The present measurements do not by themselves distinguish covalent attachment to the mineral from polymer deposition, coprecipitation, or other forms of intimate organo-mineral association. The FTC products are dominated by aromatic C–C bonds rather than by weaker ether linkages, consistent with radical coupling as the polymerization mechanism.^45^

This carbon stabilization is mechanistically coupled to manganese redox chemistry. Mn reduction and carbon accumulation proceed concomitantly under FTC. Radical-mediated catechol coupling generates increasingly mineral-associated products as the Mn-bearing solid becomes more reduced. The pronounced morphological changes observed by TEM indicate substantial alteration of the birnessite solid during FTC. However, the new reflection at 31.7° 2θ cannot be uniquely assigned to a secondary Mn phase.^46^

Birnessite reduction by organic reductants can proceed through reductive dissolution with Mn^2+^ release:

1 $${\mathrm{MnO}}_{2}\left(s\right)+{4H}^{+}\left(\mathrm{aq}\right)+{2e}^{-}\to {\mathrm{Mn}}^{2+}\left(\mathrm{aq}\right)+{2H}_{2}O$$

Under ambient aqueous conditions, Mn^2+^ release commonly accompanies reduction by organic reductants.^46^ Under FTC, XPS additionally shows substantial accumulation of Mn­(III) within the solid phase, although its resulting structure cannot be resolved from the present diffraction data.

Multiple environmental controls support freeze concentration as the operative driver. The liquid/supercooled temperature series shows that cooling alone suppresses catechol depletion, whereas the salinity dependence provides independent evidence of a freezing-related concentration effect. At higher initial salinity, the freeze-concentration factor is smaller, resulting in less enrichment of dissolved reactants within LIBs and a correspondingly weaker FTC effect. FTC samples also exhibit higher post-thaw pH values than RT samples, consistent with a different net proton balance during the freeze–thaw sequence that may partly reflect the greater extent of Mn reduction under FTC.^47–50^ However, because pH was measured only after complete thawing, these data do not establish the *in situ* pH or proton balance within the LIBs. The extension of the mechanism to preformed soluble intermediates further broadens its relevance beyond a single model compound to the natural dissolved organic carbon pool.^47–50^

## 5 Environmental Implications

The results define a previously unrecognized abiotic carbon-stabilization pathway in frozen environments. The attenuation of the mechanism at high salinity confines its greatest relevance to freshwater and low-salinity frozen environments, which encompass continental permafrost, seasonally frozen soils, frozen lakes and wetlands, and glacial systems across high-latitude and alpine regions.^29,51,52^ The broad substrate scope, extending from fresh monomers to preformed humic substances, suggests that the process could capture dissolved organic matter throughout the seasonal cycle, from plant-derived polyphenols released during autumn litter decomposition to humic substances accumulated in summer pore waters.

Dissolved phenolic monomers in natural soil solutions generally occur at submicromolar to low-micromolar concentrations, although higher concentrations can occur in organic-rich horizons and litter leachates.^26,27^ Reactive Mn phases are widespread in soils, with total reactive Mn concentrations commonly reported on a soil-mass basis of approximately 0.1–2.5 g kg^–1^ and locally reaching several g kg^–1^.^53,54^ These soil concentrations cannot be translated directly into the mineral loading used here without knowledge of local soil:water ratios, reactive mineral surface area, and organic-matter partitioning. The experimental catechol concentration is therefore higher than that of many natural soil waters. Freezing can, nevertheless, enrich dissolved solutes substantially within LIBs, providing a mechanism by which dilute environmental substrates may experience much higher local concentrations during freezing. At lower initial substrate concentrations, reaction rates, product yields, and the extent of polymer growth may differ from those observed here. Accordingly, the present results establish a mechanistic proof of concept for freeze-induced transfer of dissolved phenolic carbon into mineral-associated products, while the magnitude and environmental importance of this pathway remain to be quantified under natural conditions.

Current conceptual and numerical frameworks for soil carbon persistence attribute carbon stabilization primarily to microbial transformation and mineral sorption, largely neglecting freeze–thaw dynamics.^55–58^ Yet, this abiotic pathway could operate precisely when biological activity is suppressed by freezing. The two mechanisms are complementary, rather than competing. Microbial transformation generates dissolved organic substrates during thaw periods, while freeze concentration may promote their transfer into persistent mineral-associated forms during freezing. This creates a potential stabilization cascade across the annual freeze–thaw cycle in the permafrost and seasonally frozen environments that store approximately half of the global soil carbon.

Field validation will be essential to quantify the contribution of this pathway under natural conditions, where mineral diversity, organic matter complexity, and variable freeze–thaw regimes may modulate its importance. Nonetheless, the mechanistic framework established here provides a foundation for incorporating ice-mediated carbon stabilization into predictive models of permafrost carbon dynamics under a changing climate.

## Supplementary Material



## Notes

Activation energy determination (Arrhenius analysis) and detailed analytical protocols for UV–vis spectrophotometry, HPLC, LC-MS, fluorescence EEM-PARAFAC modeling, FTIR spectroscopy, XPS, and thermogravimetric analysis coupled with CO_2_ detection; Raman reference spectra of catechol and birnessite (Figure S1); control experiments without birnessite, confirming mineral-mediated reactivity (Figure S2); elemental carbon analysis of FTC and RT solids (Figure S3); TOC mass balance by reductive dissolution (Figure S4); headspace CO_2_ monitoring during FTC (Figure S5); time-resolved fluorescence EEM spectra under RT and FTC at 1, 30, and 100 mM NaCl (Figures S6–S11); thermogravimetric and derivative curves under an oxygen atmosphere (Figure S12); organic carbon stability tests under acid, alkaline, and NaOCl extraction (Figure S13); high-resolution XPS spectra of C 1s and O 1s regions (Figure S14); FTIR spectra of birnessite and reaction products (Figure S15); XPS spectra of the Mn 2p_3_/_2_ region with peak deconvolution (Figure S16); FTIR spectra showing Mn–O stretching frequency shifts (Figure S17); pH trajectories at initial pH 5 and 9 (Figure S18); PARAFAC model validation and component identification (Figures S19–S21); time-dependent variations of PARAFAC Components 1 and 2 (Figure S22); additional UV–vis data at pH 7 under varying NaCl concentrations (Figure S23); time-resolved UV–vis and PARAFAC fluorescence data showing FTC–RT differences at initial pH 5 and 9 (Figures S24–S29); UV–vis evidence for secondary processing of preformed soluble products by FTC with fresh birnessite (Figure S30) (PDF)
