# Supplementary material for: Ice Converts Phenols into Persistent Mineral-Bound Polymers
Source: Environ Sci Technol. 2026 Oct 14;60(42):30031–40. doi: 10.1021/acs.est.6c05553 (PMC13718625; doi:10.1021/acs.est.6c05553)
Supplement: Supplementary file 1 [file es6c05553_si_001.pdf]

## Supporting Information for

# Ice converts phenols into persistent mineral-bound polymers

*Yang Rong[1,2], Tao Luo,[1,3], Tao Chen[1], Tra My Bui Thi[1], Khalil Hanna[1], Jean-François Boily[1,*]*

1. Department of Chemistry, Umeå University SE 901 87 Umeå, Sweden

2. Université de Rennes, École Nationale Supérieure de Chimie de Rennes, CNRS, ISCR-UMR 6226, F-35000 Rennes, France.

3. College of Chemistry, Chemical Engineering and Materials Science, Shandong Normal University, Jinan 250014, China

*Jean-François Boily
**Email:** jean-francois.boily@umu.se

**Table of Contents**


1. Supplementary Methods ........ 3

1.1 Activation Energy Determination ........ 3

1.2 Analytical methods ........ 3

1.2.1 Ultraviolet-Visible (UV-vis) Spectrophotometry ........ 3

1.2.2 High-Performance Liquid Chromatography (HPLC) ........ 3

1.2.3 Liquid Chromatography-Mass Spectrometry (LC-MS) ........ 3

1.2.4 Fluorescence Excitation-Emission Matrices and PARAFAC Analysis ........ 3

1.2.5 Fourier Transform Infrared (FTIR) Spectroscopy ........ 4

1.2.6 X-ray Photoelectron Spectroscopy (XPS) ........ 4

1.2.7 Thermogravimetric Analysis (TGA) ........ 4

1.3 Data treatment and numerical analyses ........ 4

1.3.1 Composition Analysis by EEM-PARAFAC ........ 4

1.3.2 Activation Energies for Catechol-Birnessite Reactions ........ 5

2. Supplementary Figures ........ 6

3. Supplementary Tables ........ 26

4. References ........ 29

## 1. Supplementary Methods

**1.1** Activation Energy Determination

Reactions were carried out under controlled temperature conditions using either a temperature-controlled room (20 ± 1 °C), a water bath (10 ± 0.1 °C), or an ethanol bath (4, 0, and −5 °C; ± 0.01 °C). Batch reactions were performed into Falcon® conical centrifuge tubes containing 50 μM catechol and 50 mg $L^{-1}$ birnessite. After preparation, samples were incubated at the target temperature and collected at predetermined time intervals for kinetic analysis. The temperature-dependent rate constants obtained from these experiments were used to construct an Arrhenius plot and calculate the apparent activation energy.

### 1.2 Analytical methods

*1.2.1 Ultraviolet-Visible (UV-vis) Spectrophotometry*

Organic reaction products were characterized using UV-vis spectra of filtered samples. Spectra were acquired over 200–700 nm using a quartz cuvette with 10 mm optical path length on a Cary-50 UV-vis spectrophotometer (Varian).

*1.2.2 High-Performance Liquid Chromatography (HPLC)*

Aqueous catechol concentrations were determined using HPLC with a Waters 600 controller equipped with a reversed-phase C18 column (250 × 4.6 mm, 5 μm) and UV detector (Waters 2489) set at 275 nm. The mobile phase consisted of acetonitrile/water (20:80, v/v) with 0.1% formic acid.

*1.2.3 Liquid Chromatography-Mass Spectrometry (LC-MS)*

LC-MS analyses were performed on an Agilent Technologies 6230 Time-of-Flight (TOF) LC/MS system with C18 column. Chromatographic separation used gradient elution at 0.4 mL/min with a 12-min total run time. Data acquisition was performed in full-scan mode.

*1.2.4 Fluorescence Excitation-Emission Matrices and PARAFAC Analysis*

EEM-PARAFAC(1) was used for DOM characterization (see SI for model validation). Fluorescence EEMs were measured with a luminescence spectrometer(1) (xenon source, Perkin-Elmer) by scanning emission from 250 to 600 nm at 0.5 nm increments while stepping through excitation wavelengths from 220 to 500 nm at 2 nm intervals. Excitation and emission slits were 10 nm and 5 nm, respectively; scanning speed was 1000 nm/min. Milli-Q water (18.2 MΩ·cm) spectra were subtracted as blanks. Standard corrections were applied: first- and second-order Rayleigh scattering, Raman scattering subtraction, Raman normalization, and inner filter effects.(1)

PARAFAC modeling was performed using MATLAB (MathWorks) with the DOMFluor Toolbox (http://www.models.life.ku.dk). The toolbox uses the N-way toolbox as a PARAFAC engine and incorporates FDOMcorr for improved dataset manipulation, visualization, and hypothesis testing during model validation.

*1.2.5 Fourier Transform Infrared (FTIR) Spectroscopy*

FTIR spectroscopy identified Mn (oxy)(hydr)oxides and carbon species. Spectra were recorded in vacuo (<2 hPa) using an ATR accessory (Platinum, Bruker) with samples pressed by anvil. Each spectrum represents 100 scans collected over 400–4500 $cm^{-1}$ at 2 $cm^{-1}$ resolution with 10 kHz mirror scanning rate on a Bruker Vertex 70/V spectrometer equipped with DLaTGS detector.

*1.2.6 X-ray Photoelectron Spectroscopy (XPS)*

Surface manganese oxidation states were determined by XPS. Solid-phase materials from batch experiments were rinsed thoroughly with Milli-Q water (18.2 MΩ·cm) and dried under $N_2$. XPS measurements used a Kratos Axis Ultra spectrometer with monochromatic Al Kα radiation (1486.7 eV) at 10 mA and 15 kV. Manganese oxidation states were resolved through peak deconvolution of the Mn $2p_{3/2}$ region following Ilton et al.(2) Spectral fitting used CasaXPS software with Shirley background applied uniformly.

*1.2.7 Thermogravimetric Analysis (TGA)*

Carbon oxidation at different temperatures was investigated using TGA (TA Instruments TGA 5500). Samples were evenly distributed on aluminum pans (⌀ 8 mm, 1 mm edge height) and heated in an infrared furnace under controlled gas flow (100 ± 0.02 mL/min synthetic gas: 80% $N_2$ + 20% $O_2$) at 10°C/min. Evolved $CO_2$ was quantified in real time using a dual-cell differential gas analyzer (LI-7000 $CO_2$/$H_2O$ Analyzer, LI-COR Biosciences) employing a dichroic beam splitter and independent detectors for simultaneous $CO_2$ and $H_2O$ measurement under continuous flow with automatic pressure compensation.

**1.3 Data treatment and numerical analyses**

*1.3.1 Composition Analysis by EEM-PARAFAC*

PARAFAC modeling of the entire reaction process identified a three-component solution, with the three-component model supported by core consistency and explained-variance analyses (Figure S19). The resolved fluorescence components are shown in Figure S20. Component 1 corresponds to catechol; Component 2 is most plausibly an electron-transfer product derived from catechol; Component 3, based on comparison with spectral databases and literature, is assigned as humic-like acid (HLA) (Figures S20 and S21). The time-dependent variations of Components 1 and 2 are presented in Figure S22. Component 1 decreases steadily over the reaction, whereas Component 2 exhibits a transient increase followed by decline, consistent with its role as an intermediate formed from catechol and subsequently consumed through further polymerization. Component 3 increases continuously, reflecting progressive HLA accumulation. Component 3 (HLA) is certainly a mixture, but no further separation was attempted because fluorescence spectroscopy does not permit such fine resolution. The main text focuses primarily on the third component. The original EEM spectra are shown in Figures S6–S11.

*1.3.2 Activation Energies for Catechol-Birnessite Reactions*

To determine the activation energy of the catechol-birnessite reaction, kinetic experiments were conducted at different temperatures (20, 10, 4, 0, −5°C). Activation energy was calculated from the temperature dependence of the reaction rate constant using the Arrhenius equation:

$$k = A \cdot e^{(-Ea/RT)} \qquad \text{(S1)}$$

where k is the rate constant, T is absolute temperature, A is the pre-exponential factor, $E_a$ is the molar activation energy, and R is the universal gas constant.

The catechol-birnessite reaction was fitted to a pseudo-first-order kinetic model:

$$\ln\left(\frac{C_t}{C_0}\right) = -kt \qquad \text{(S2)}$$

where $C_0$ and $C_t$ are catechol concentrations at initial time and time t, respectively, and k is the apparent first-order rate constant. By plotting ln k against 1/T, the activation energy was obtained from the slope ($-E_a/R$).

## 2. Supplementary Figures

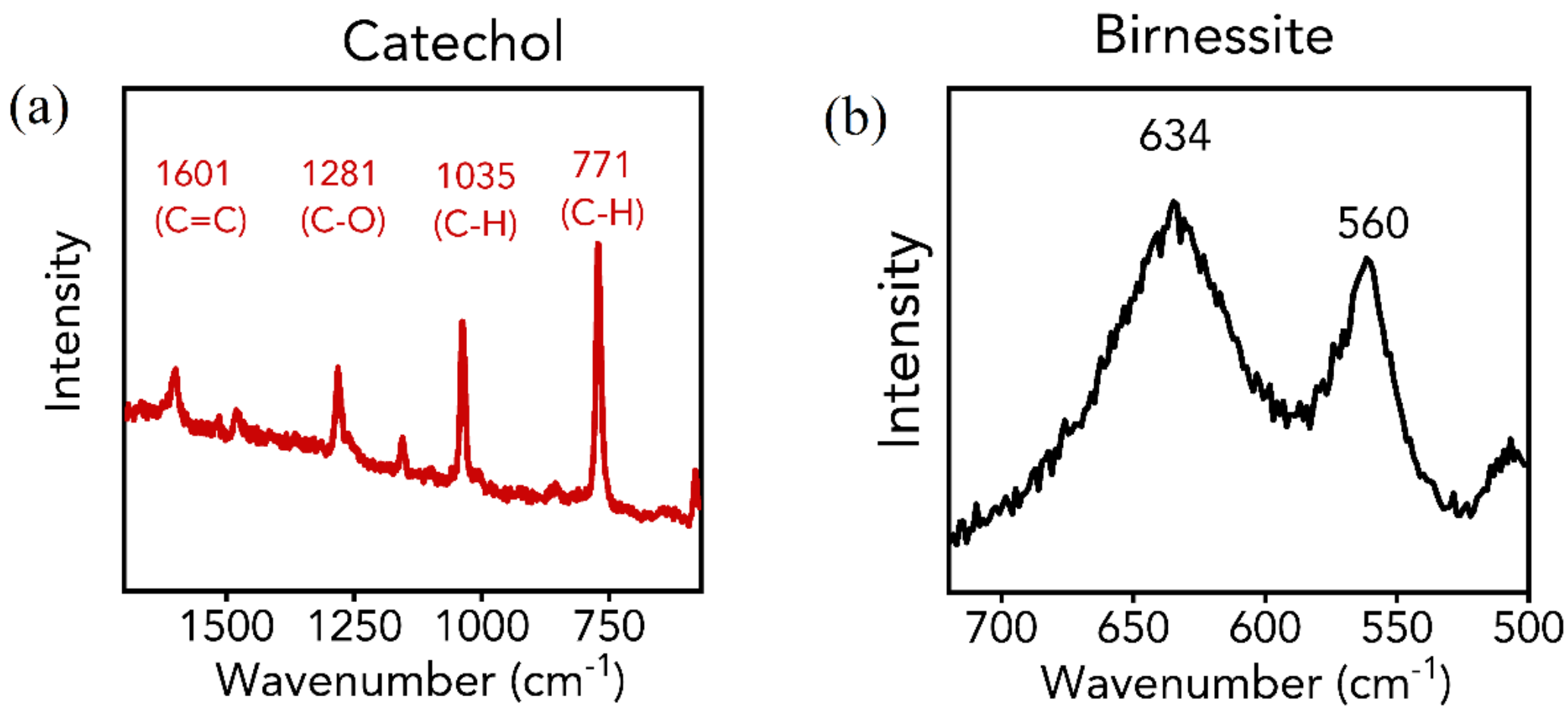


**Figure S1.** Raman spectra of catechol and birnessite reference.

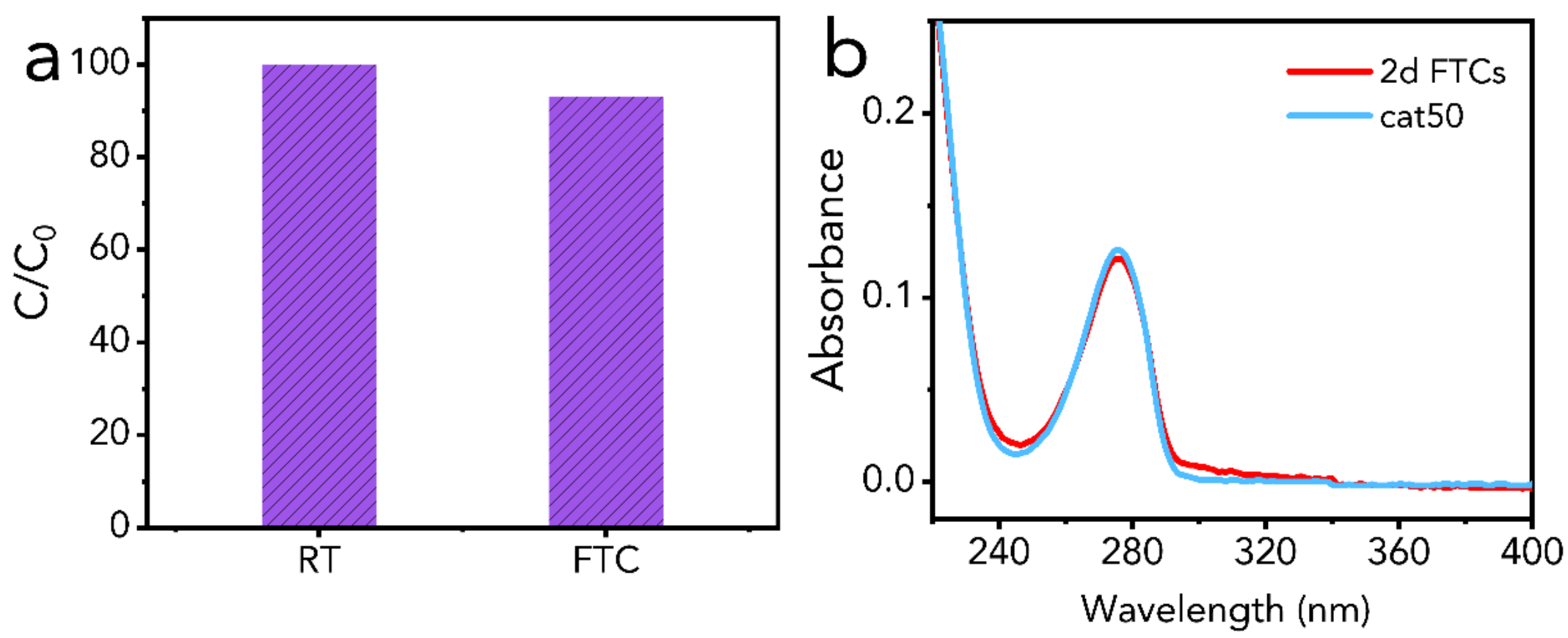


**Figure S2.** (a) Residual catechol after two days of reaction without $MnO_2$ at RT and FTCs. (b) UV-vis spectra of reaction products after two days under FTCs. Experimental conditions: $[catechol]_0$ = 50 μM; $pH_0$ = 7.0, [NaCl] = 1 mM.

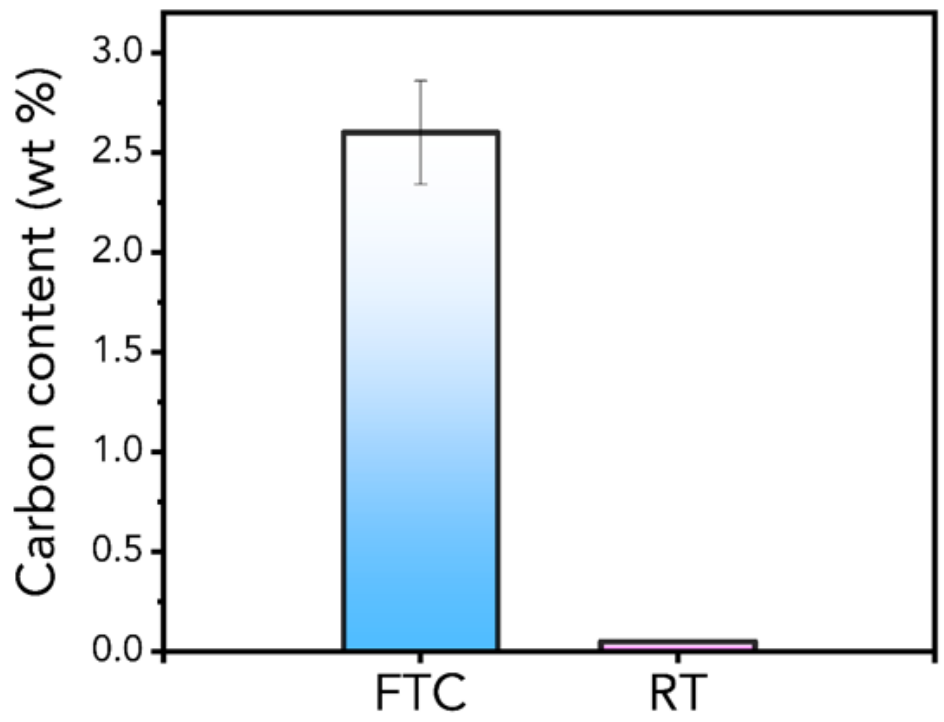


**Figure S3.** Combustion analysis of the FTC and RT solids shows substantial carbon incorporation under FTC (C = 2.6 wt%) but only trace carbon under RT (C = 0.05 wt%) Experimental conditions: [catechol]$_0$ = 500 μM, [$MnO_2$] = 500 mg $L^{-1}$, $pH_0$ = 7.0, [NaCl] = 1 mM. Solids were collected after 2 days (48 h) of reaction.

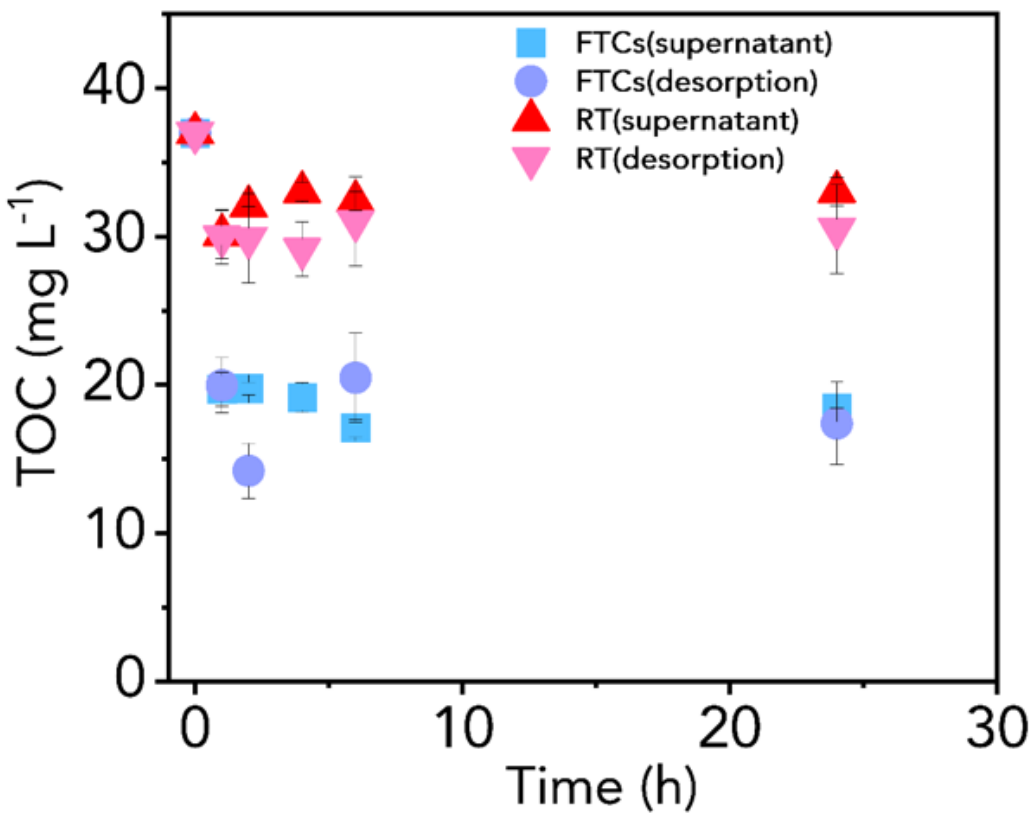


**Figure S4.** Time course of TOC change. Experimental conditions: [catechol]$_0$ = 500 μM; [$MnO_2$] = 500 mg $L^{-1}$; $pH_0$ = 7.0; [NaCl] = 1 mM.

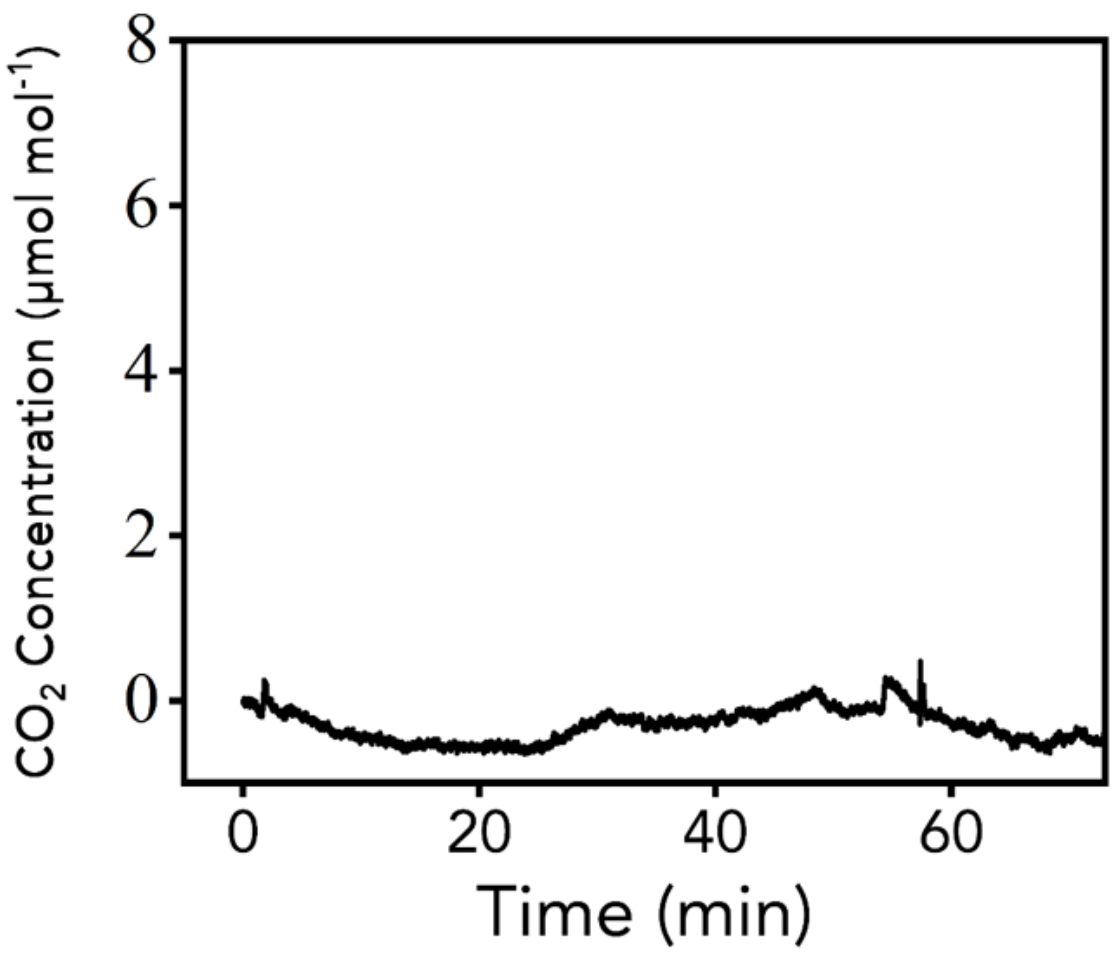


**Figure S5.** The amount of carbon dioxide released during the reaction during a freeze-thaw cycle. Experimental conditions: [catechol]$_0$ = 50 μM; [$MnO_2$] = 50 mg $L^{-1}$; $pH_0$ = 7.0; [NaCl] = 1 mM.

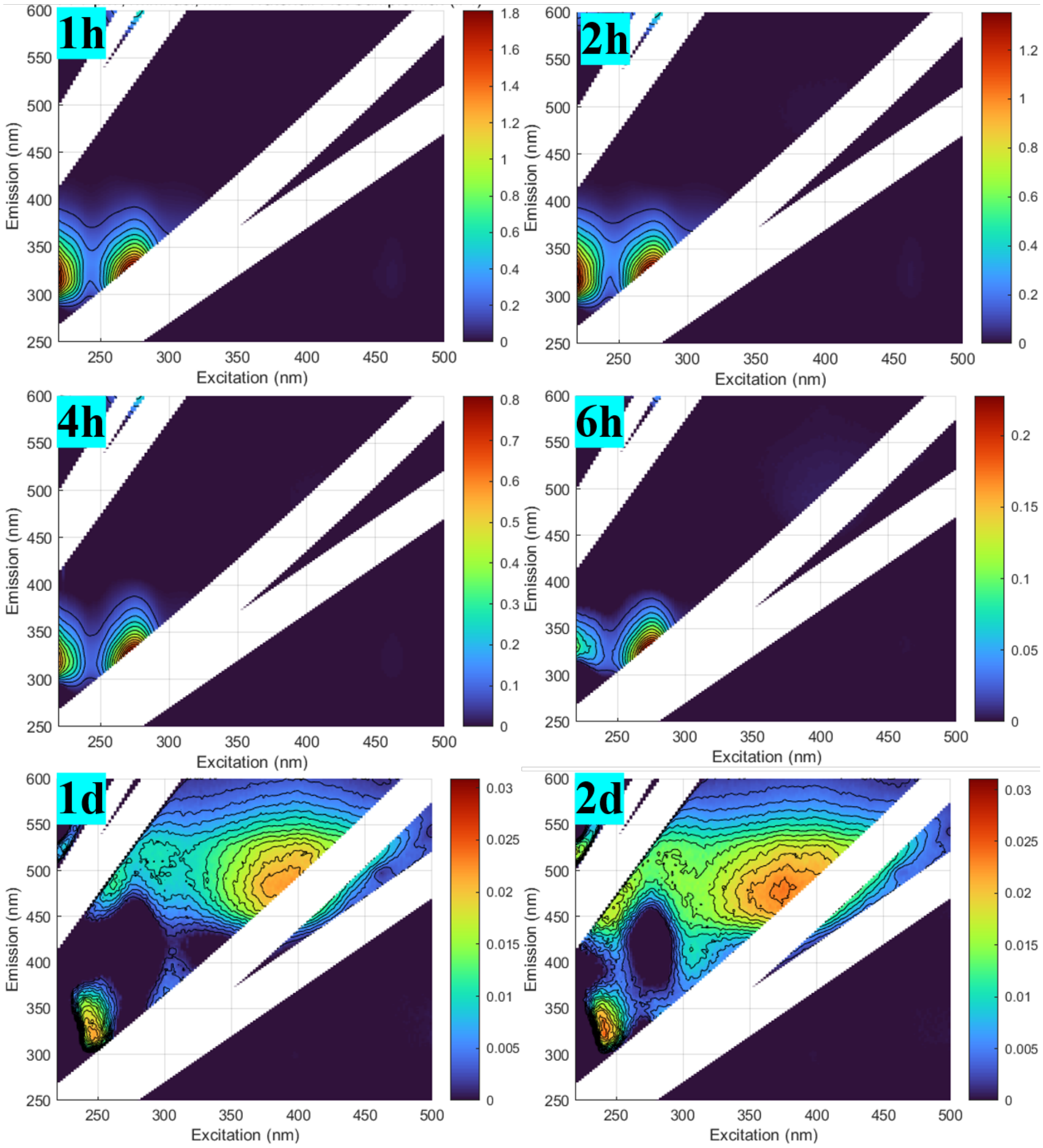


**Figure S6.** EEM spectra of reaction products over the 1 h to 2 d period at RT. Experimental conditions: $[catechol]_0$ = 50 μM; $[MnO_2]$ = 50 mg $L^{-1}$; $pH_0$ = 7.0, [NaCl] = 1 mM.

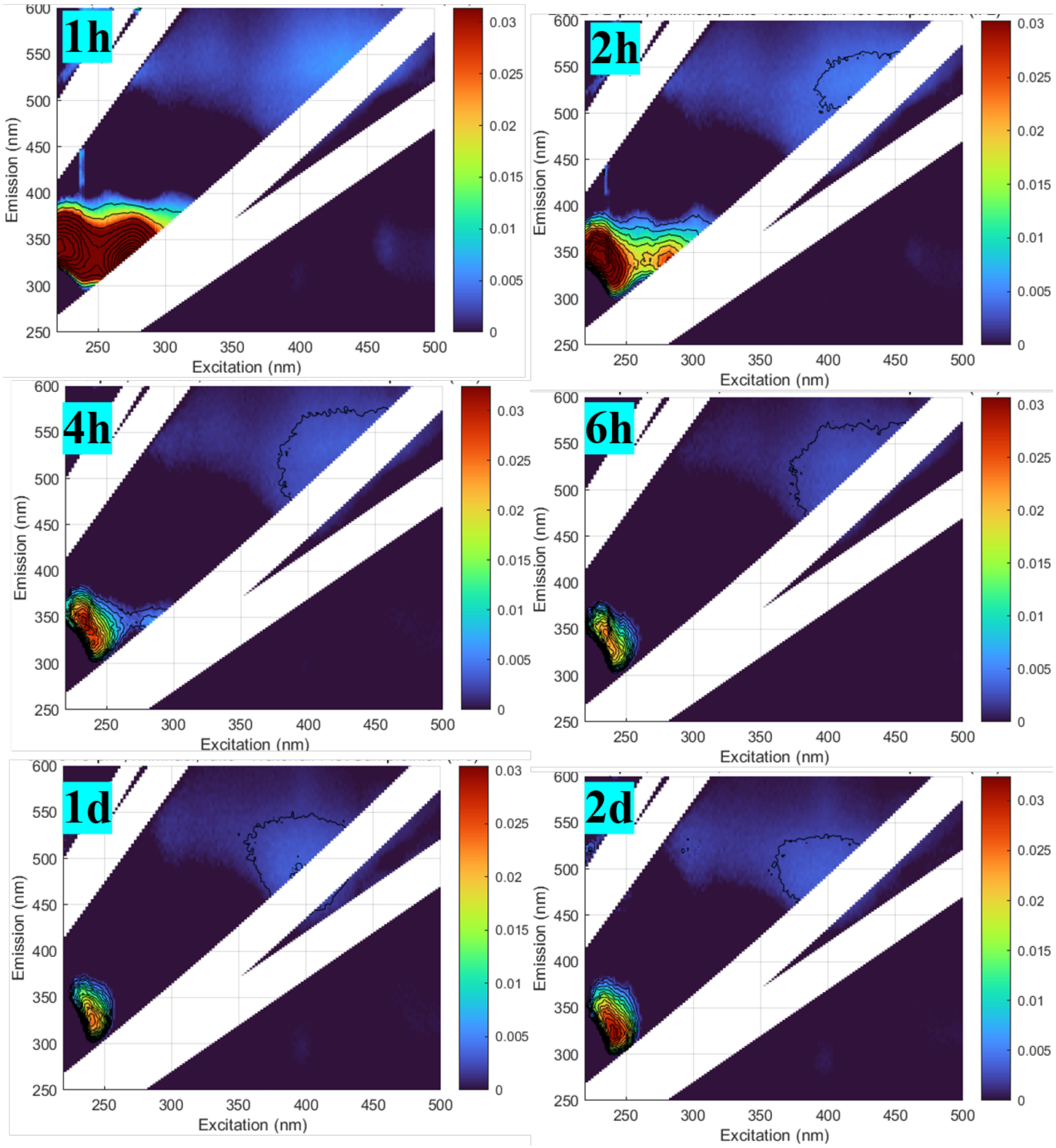


**Figure S7.** EEM spectra of reaction products over the 1 h to 2 d period at FTC. Experimental conditions: $[catechol]_0$ = 50 μM; $[MnO_2]$ = 50 mg $L^{-1}$; $pH_0$ = 7.0, [NaCl] = 1 mM.

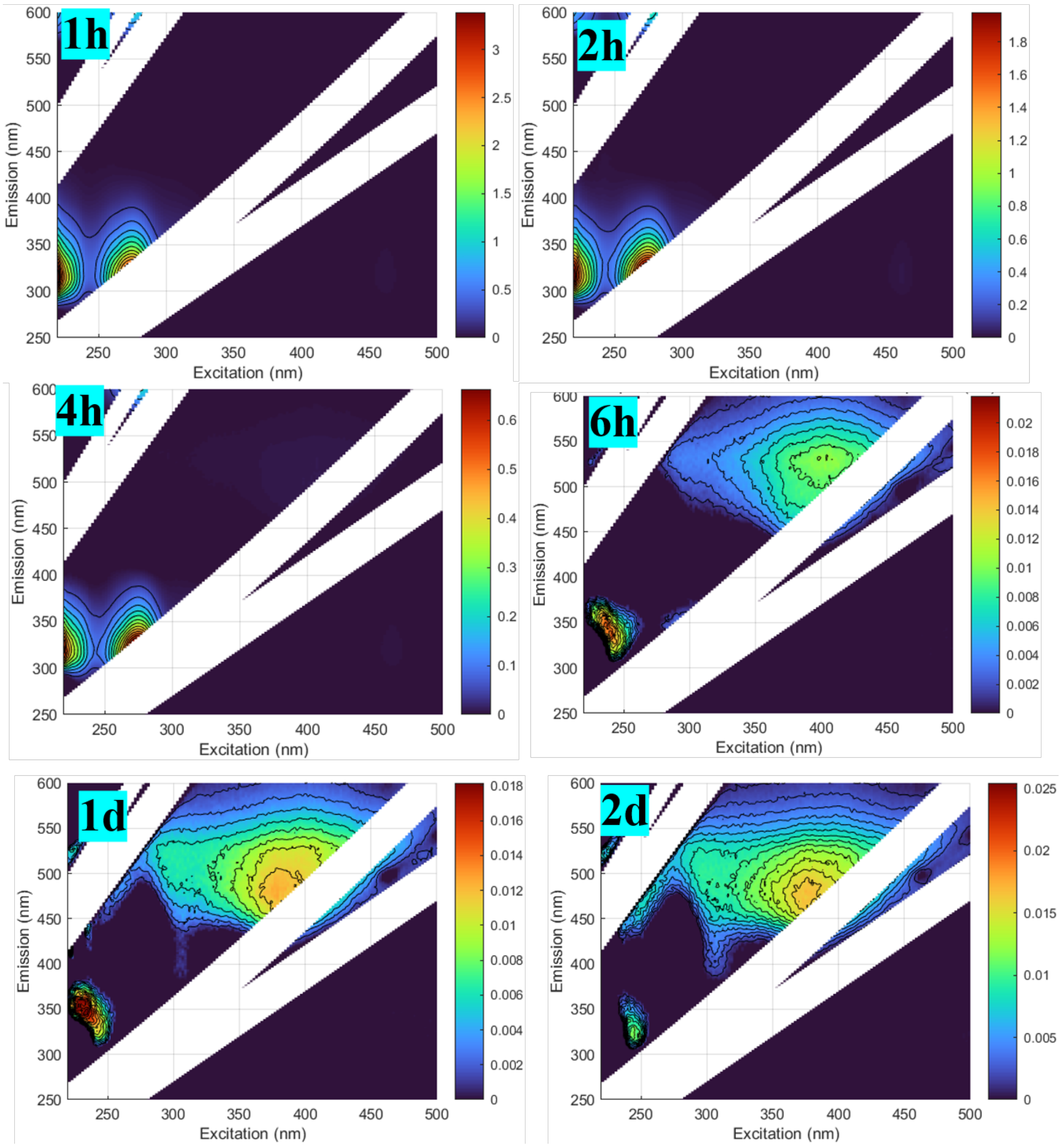


**Figure S8.** EEM spectra of reaction products over the 1 h to 2 d period at RT. Experimental conditions: $[\text{catechol}]_0$ = 50 μM; $[MnO_2]$ = 50 mg $L^{-1}$; $pH_0$ = 7.0, [NaCl] = 30 mM.

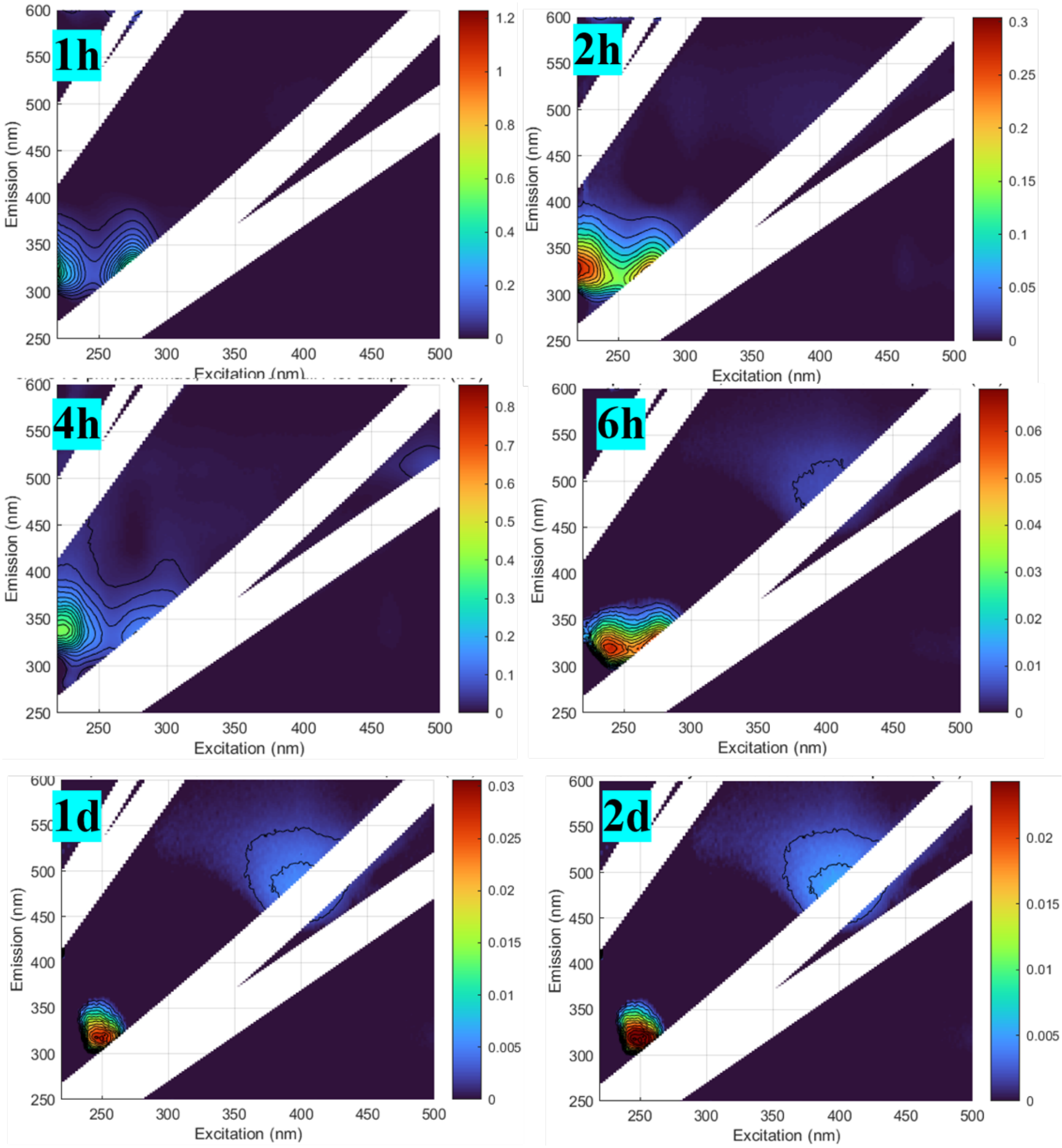


**Figure S9.** EEM spectra of reaction products over the 1 h to 2 d period at FTC. Experimental conditions: $[catechol]_0$ = 50 μM; $[MnO_2]$ = 50 mg $L^{-1}$; $pH_0$ = 7.0, [NaCl] = 30 mM.

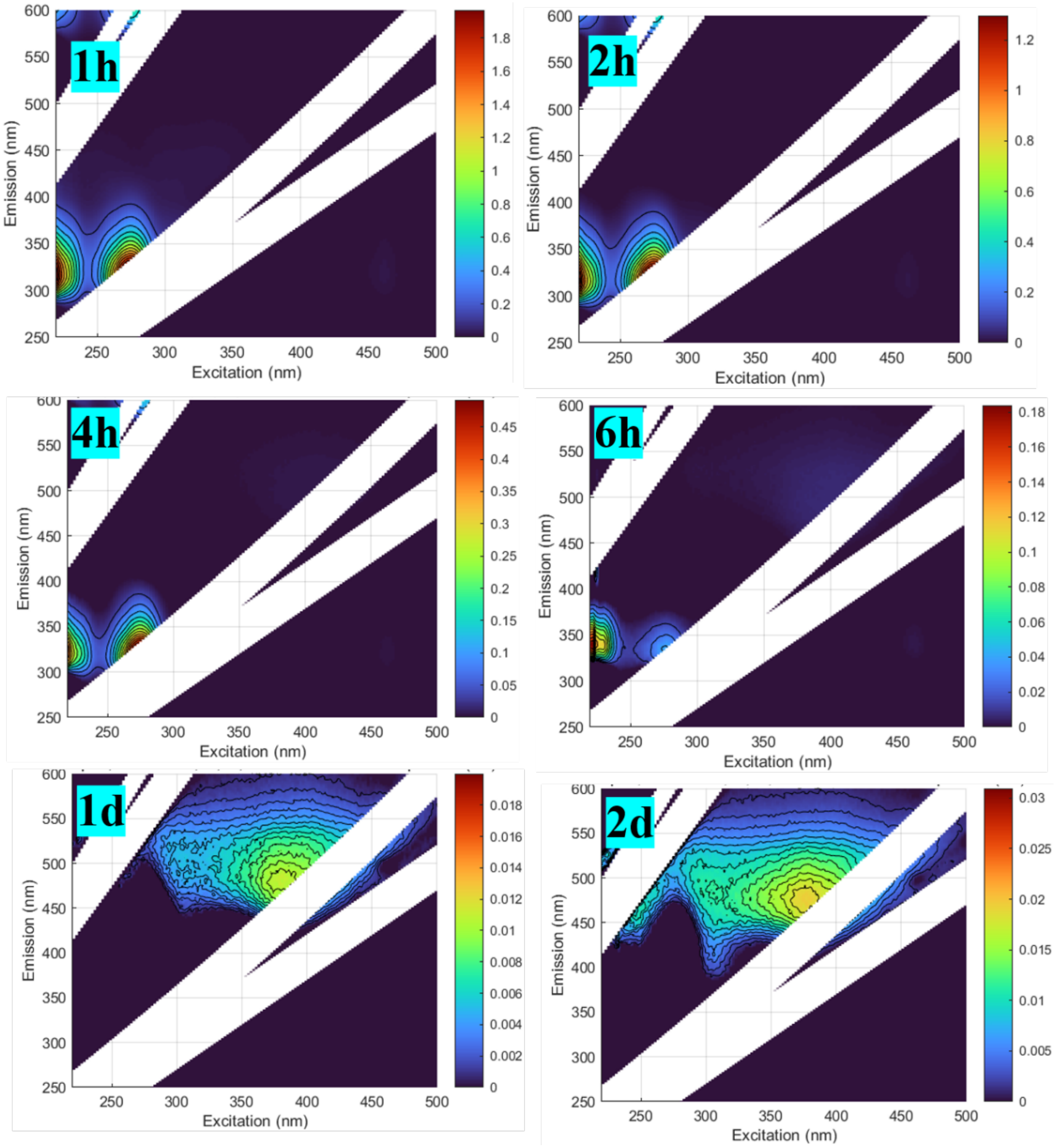


**Figure S10.** EEM spectra of reaction products over the 1 h to 2 d period at RT. Experimental conditions: $[catechol]_0$ = 50 μM; $[MnO_2]$ = 50 mg $L^{-1}$; $pH_0$ = 7.0, [NaCl] = 100 mM.

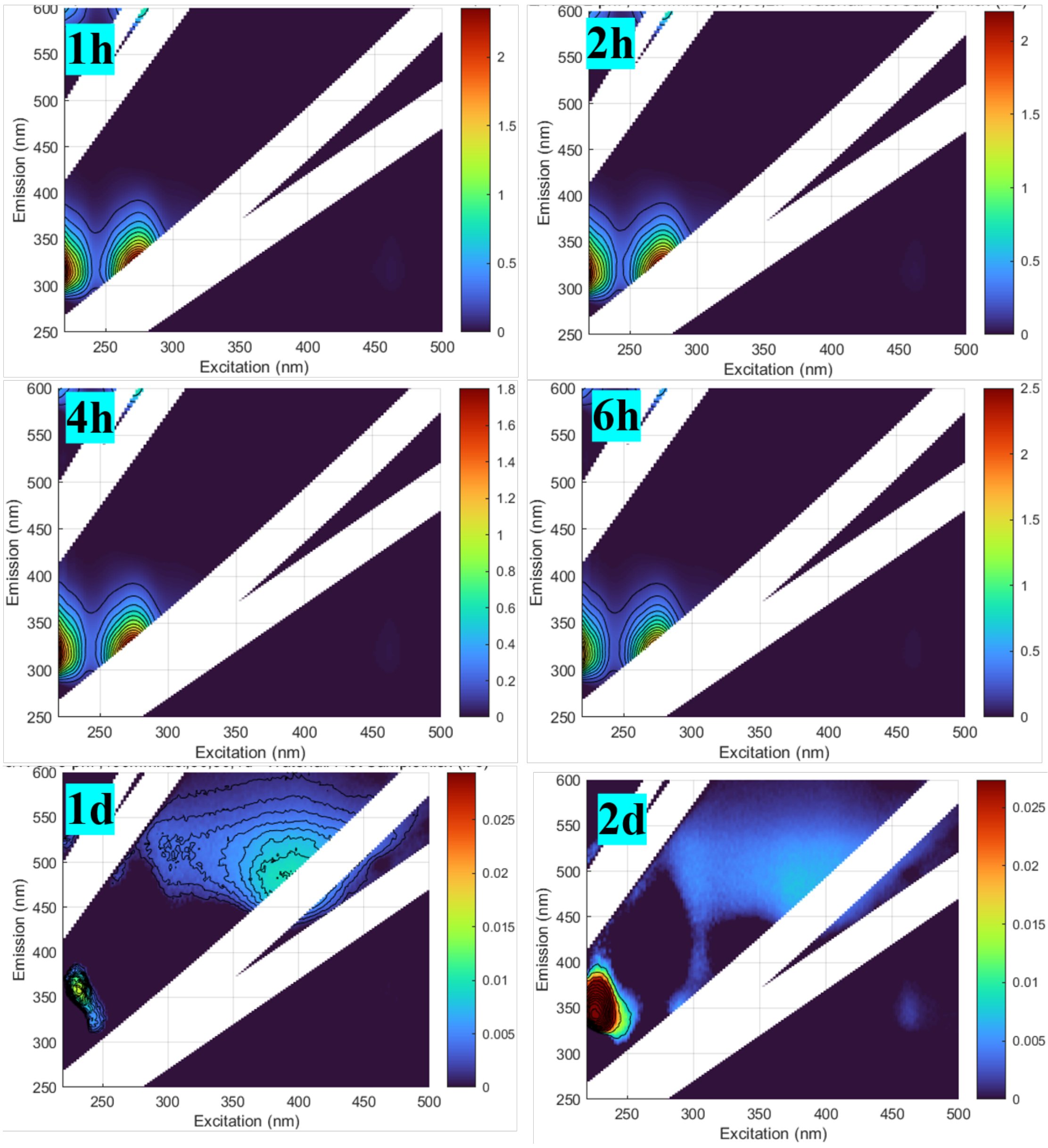


**Figure S11.** EEM spectra of reaction products over the 1 h to 2 d period at FTC. Experimental conditions: [catechol]$_0$ = 50 μM; [$MnO_2$] = 50 mg $L^{-1}$; $pH_0$ = 7.0, [NaCl] = 100 mM.

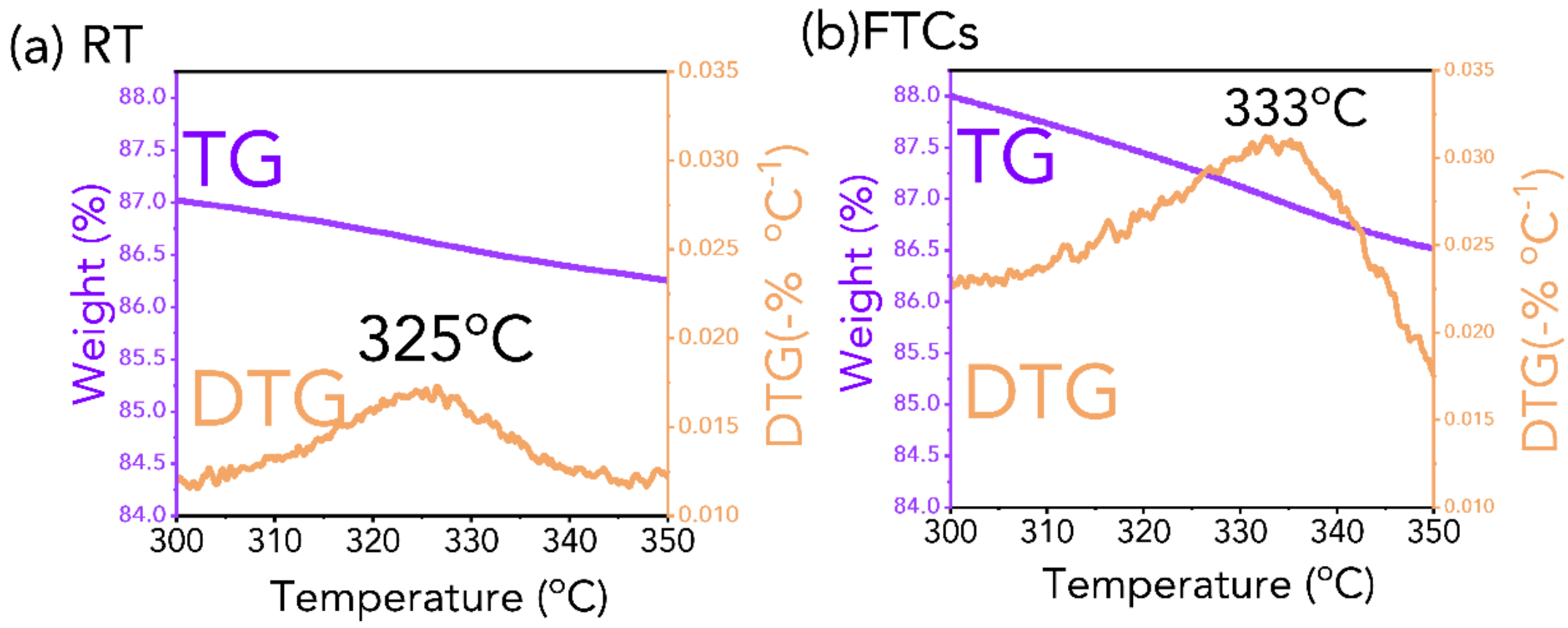


**Figure S12.** TG and DTG curves of the samples under an oxygen atmosphere. (a) RT and (b) FTCs.

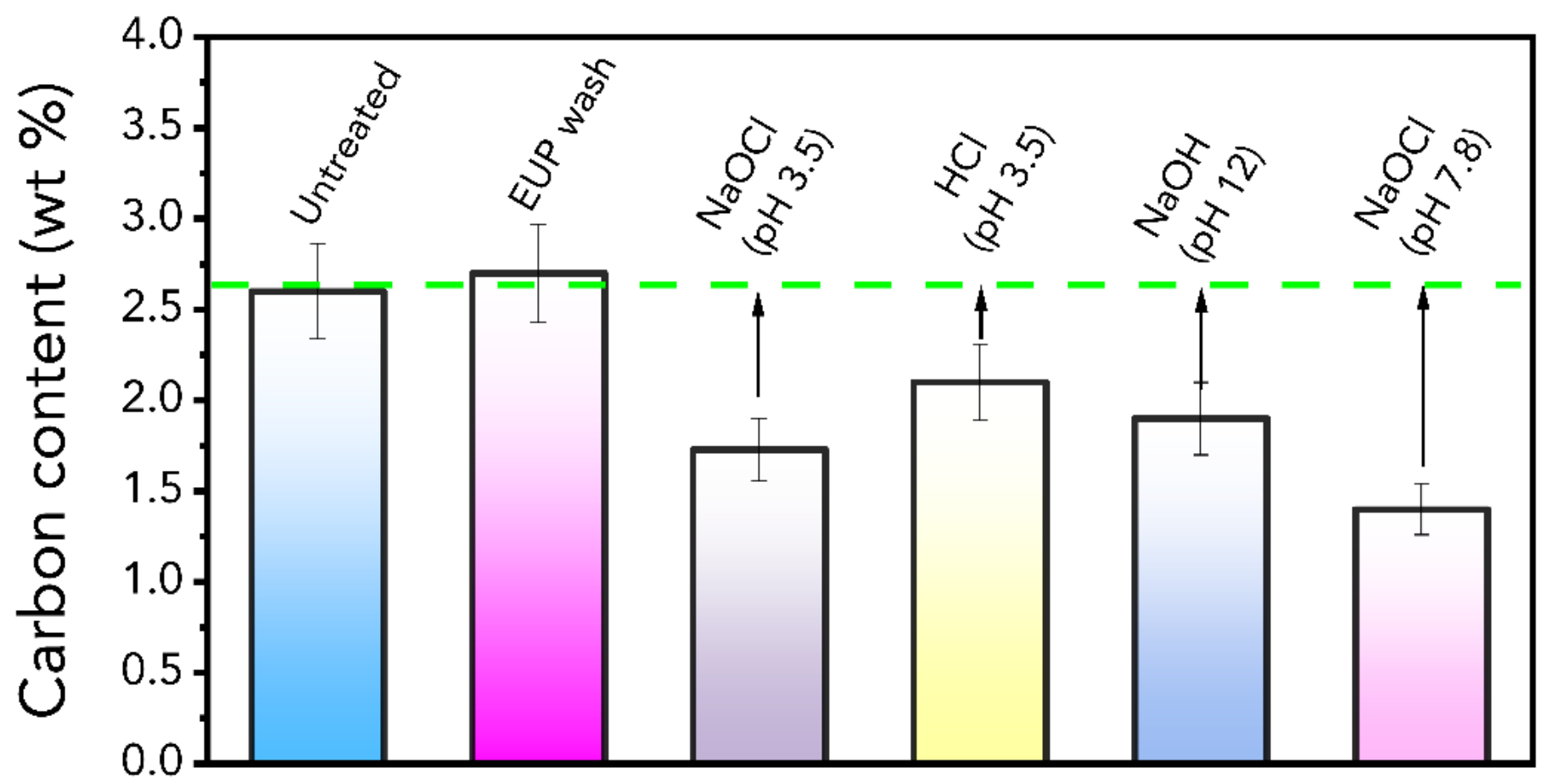


**Figure S13.** OC stability tests quantified as residual solid-phase carbon content (detected by elemental analysis): untreated FTC solid (2.6 wt%), EUP wash (2.7 wt%; within analytical uncertainty), NaOCl (pH 3.5; 1.73 wt%), HCl (pH 3.5; 2.1 wt%), NaOH (pH 12; 1.9 wt%), and NaOCl (pH 7.8; 1.4 wt%). The dashed line marks the initial carbon content of the untreated FTC solid. Error bars represent triplicate elemental analyses (mean ± SD). Conditions: birnessite–catechol reacted under FTC (same as main experiments; e.g., 500 μM catechol, 500 mg $L^{-1}$ birnessite, $pH_0$ 7, 1 mM NaCl, 2 days).

Chemical stability tests corroborated these findings. The polymeric carbon exhibited strong acid resistance at pH 3.5, retaining approximately 81% of carbon. This indicates minimal contribution from labile, low-molecular-weight products and supports the predominance of structurally condensed, mineral-associated carbon.(3, 4) High residual carbon following NaOCl oxidation at pH 3.5 (67% retained) and pH 7.8 (54% retained) demonstrates an oxidation-resistant pool. The lower retention under circumneutral conditions likely reflects enhanced solubilization of partially oxidized, carboxyl-rich compounds. Even under strongly alkaline conditions (NaOH, pH 12), 73% of the carbon was retained, demonstrating the high stability of the formed carbon against alkali-induced solubilization and disruption of organo-mineral linkages.(5) Together, these tests confirm that FTC produces chemically robust carbon resistant to conditions that would mobilize weakly bound or adsorbed organic matter.

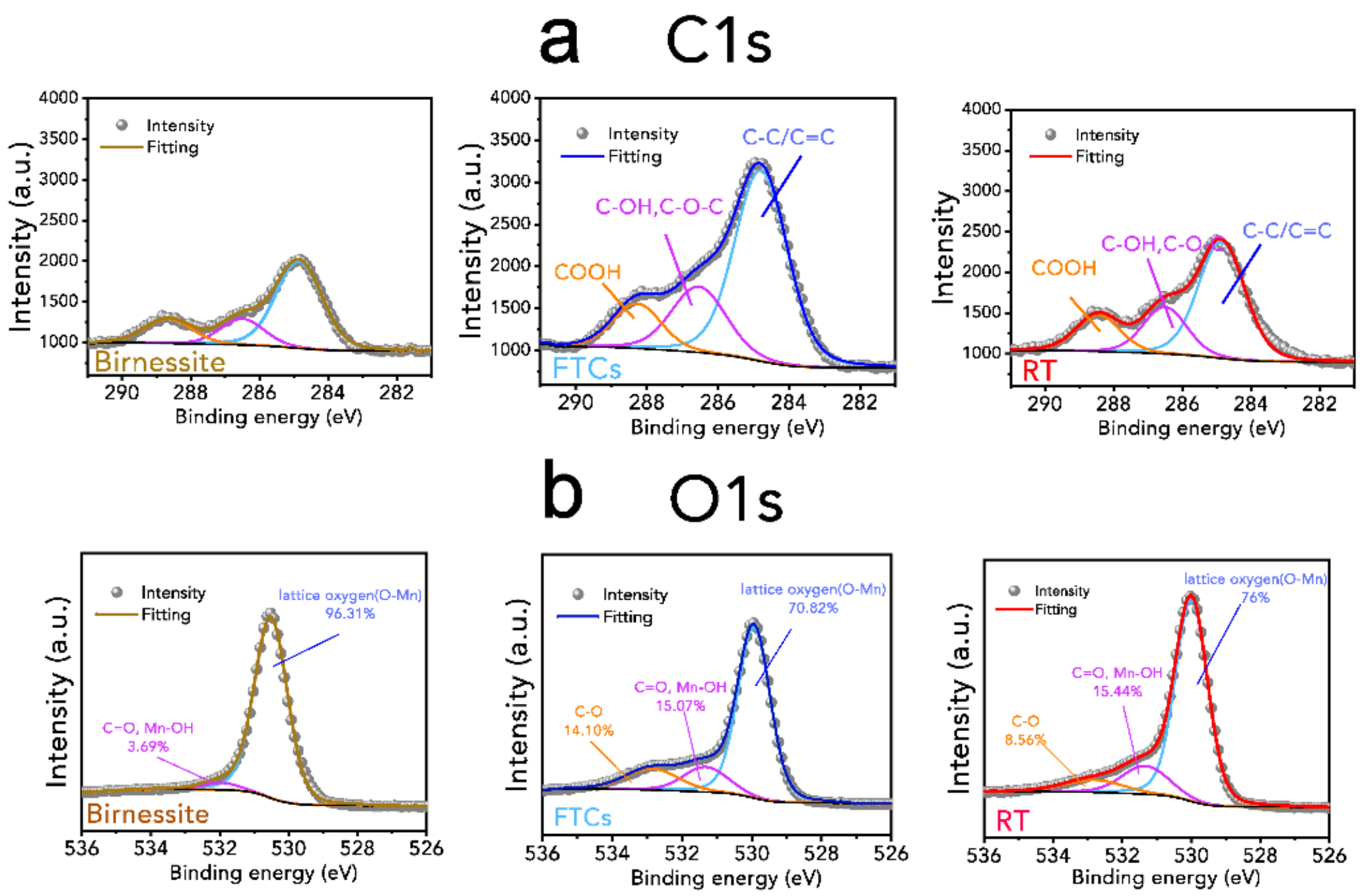


**Figure S14.** High-resolution XPS spectra of the C 1s (a) and O 1s (b) regions for pristine birnessite and solid products formed under FTC and RT conditions. Experimental conditions: $[catechol]_0$ = 500 μM; $[MnO_2]$ = 500 mg $L^{-1}$; pH0 = 7.0; [NaCl] = 1 mM.

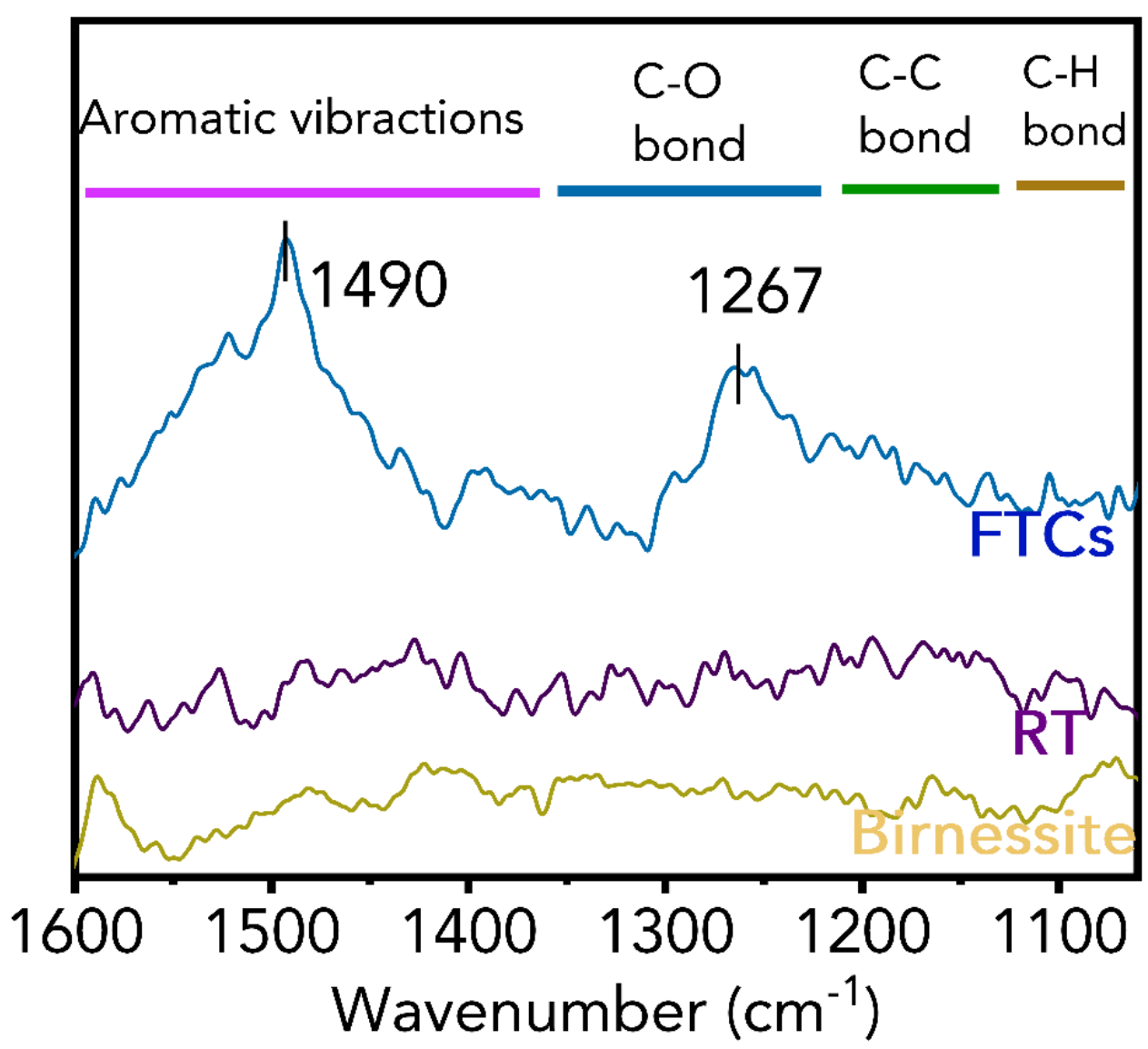


**Figure S15.** FTIR spectra of birnessite and reaction products obtained at room temperature (RT) and after freeze–thaw cycles (FTCs), illustrating the evolution of characteristic organic vibrational bands. Experimental conditions: 500 μM catechol, 500 mg $L^{-1}$ birnessite, $pH_0$ = 7.0, 1 mM NaCl.

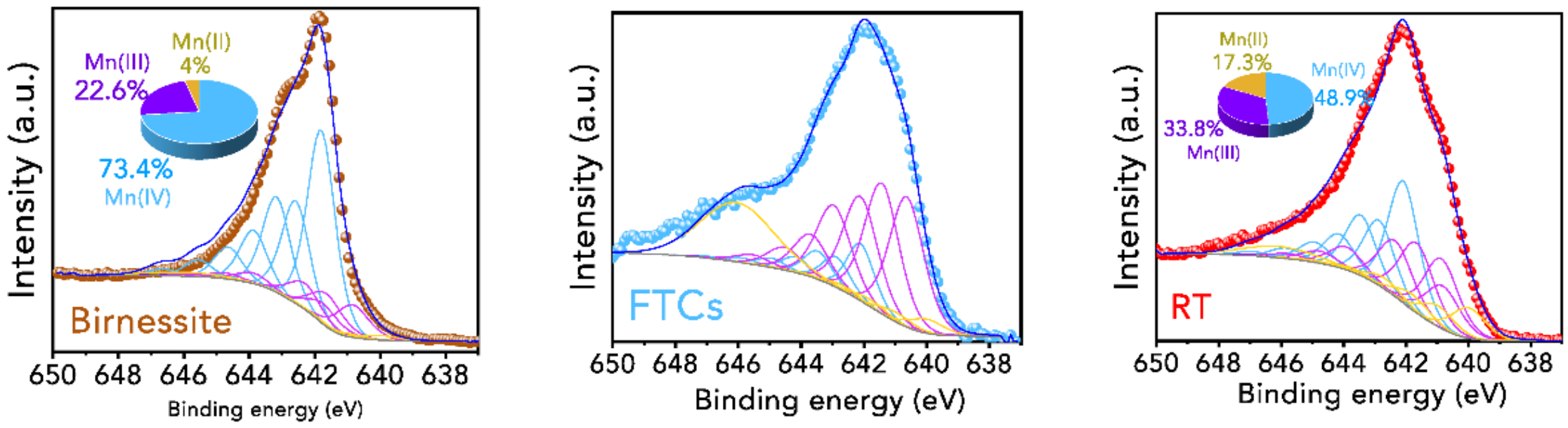


**Figure S16.** High-resolution XPS spectra of the Mn $2p_{3/2}$ region for pristine birnessite and solid products formed under FTC and RT conditions. Experimental conditions: $[\text{catechol}]_0$ = 500 μM; $[MnO_2]$ = 50 mg $L^{-1}$; pH0 = 7.0; [NaCl] = 1 mM.

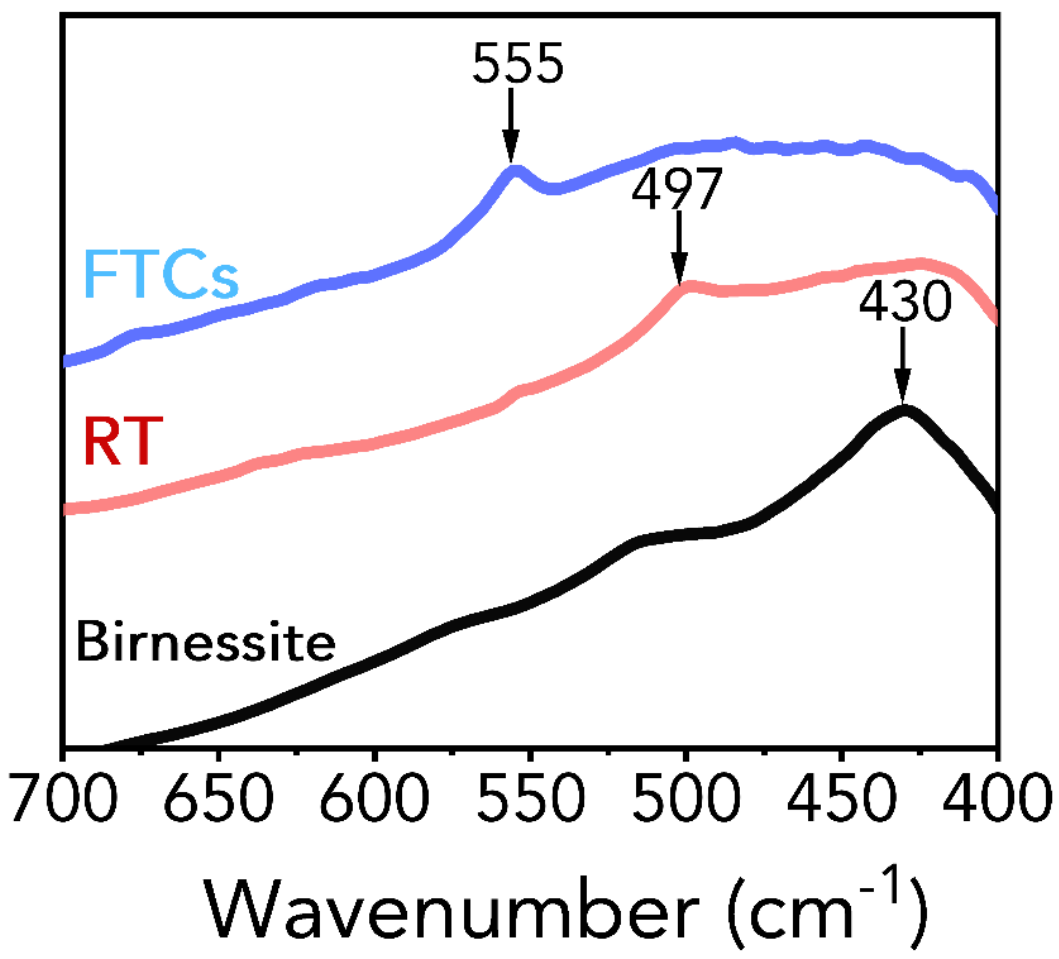


**Figure S17.** FTIR spectra of birnessite reacted with catechol at room temperature (RT) and after freeze–thaw cycles (FTCs). Experimental conditions: $[\text{catechol}]_0$ = 500 μM; $[MnO_2]$ = 50 mg $L^{-1}$; $pH_0$ = 7.0, [NaCl] = 1 mM.

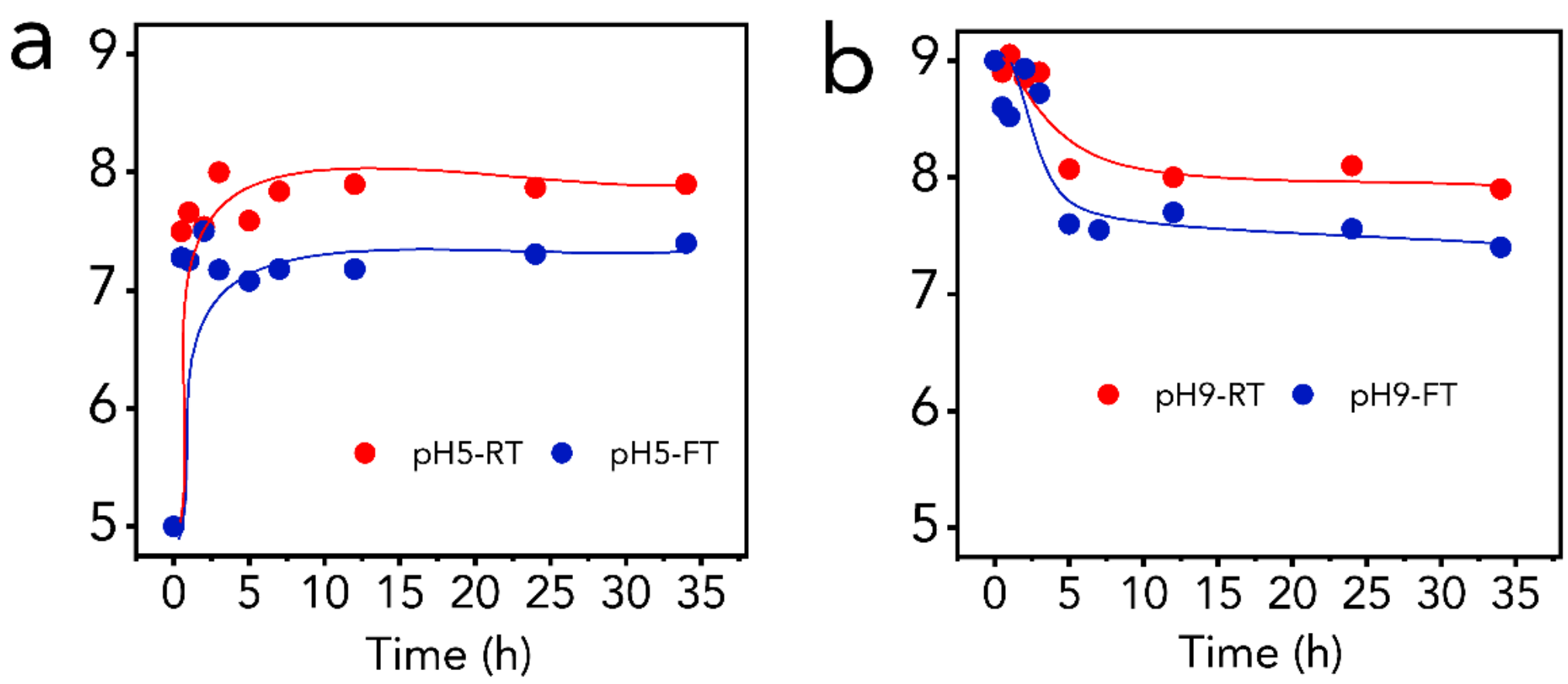


**Figure S18.** pH evolution under RT and FT conditions at initial pH values of (a) 5 and (b) 9. Experimental conditions: $[\text{catechol}]_0$ = 50 μM; $[MnO_2]$ = 50 mg $L^{-1}$; [NaCl] = 1 mM.

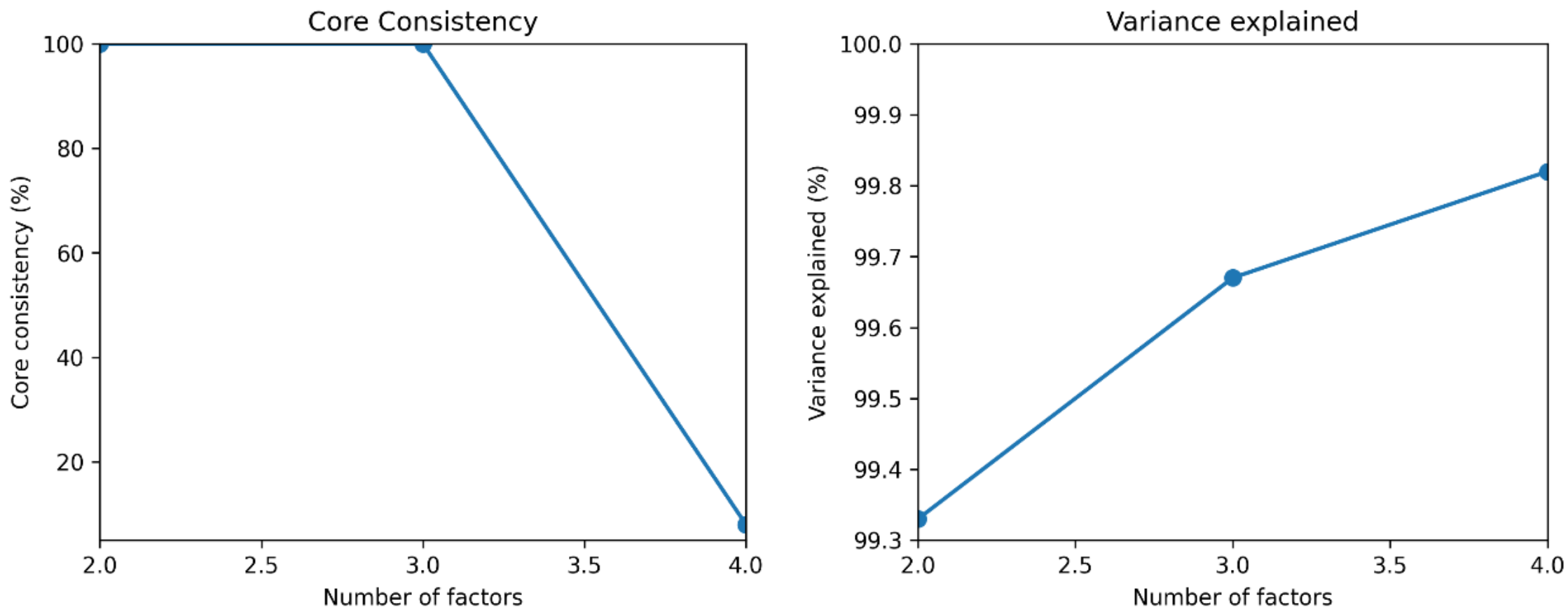


**Figure S19.** Model validation for PARAFAC analysis. (a) Core consistency diagnostic indicating that the three-component model is optimal, as the consistency remains near 100% for two and three components but drops sharply at four components. (b) Percentage of variance explained by the models, which increases slightly with the number of components, further supporting the choice of the three-component solution as both parsimonious and robust.

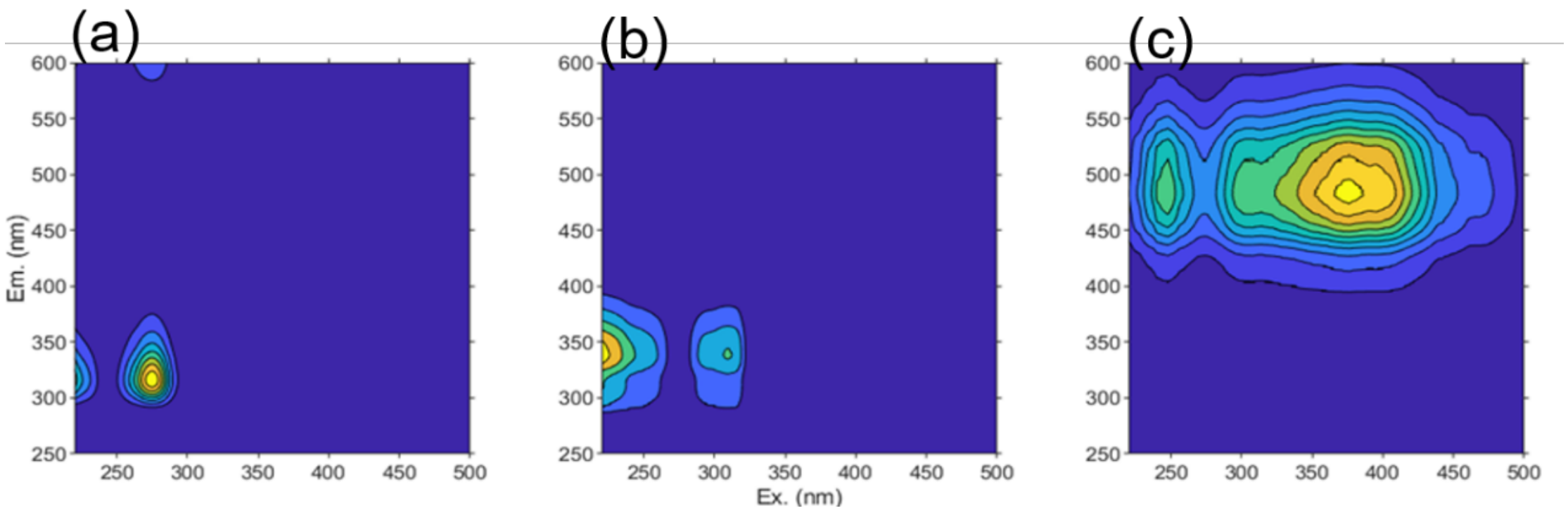


**Figure S20.** Contour plots of the excitation–emission matrices (EEMs) of the three fluorescent components resolved by the PARAFAC model. Each panel represents one component, showing distinct excitation and emission maxima: the first component (a), with a peak at Ex/Em ≈ 270/320 nm (aromatic or catechol-related), the second (b), with a peak at Ex/Em ≈ 300/340 nm (aromatic or intermediate product), and the third (c), with a broad peak at Ex/Em ≈ 340-420/450- 520 nm (humic-like substance). These components correspond to the progression from catechol to its oxidation intermediates and further polymerized humic-like products.

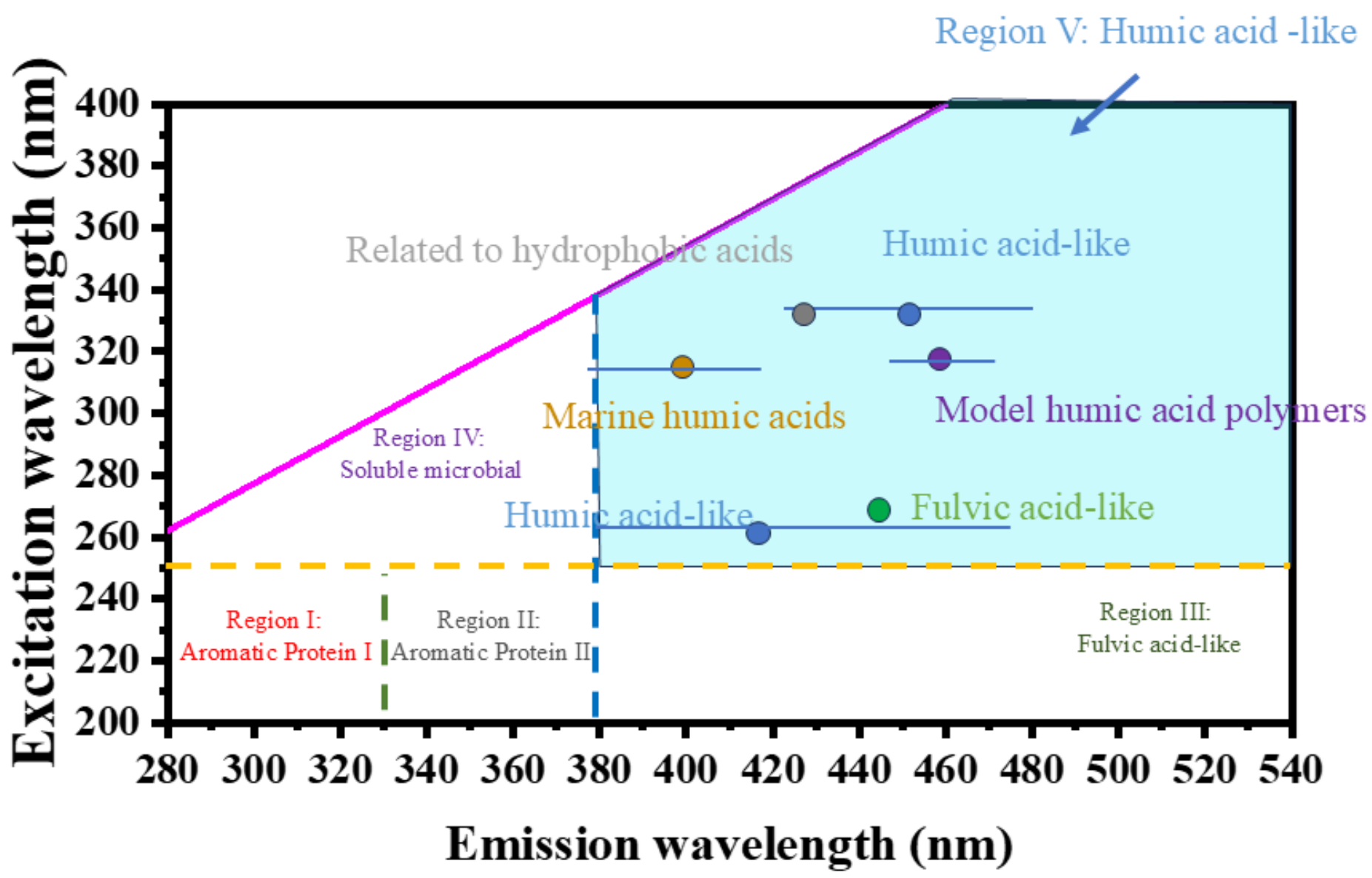


**Figure S21.** Locations of reported EEM peaks (symbols) based on literature sources, overlaid on operationally defined excitation-emission wavelength boundaries (dashed lines) that delineate five characteristic fluorescence regions: Region I (aromatic protein I), Region II (aromatic protein II), Region III (fulvic acid-like), Region IV (soluble microbial byproducts), and Region V (humic acid-like). This classification provides a reference framework for identifying dissolved organic matter components in experimental EEM fingerprints.

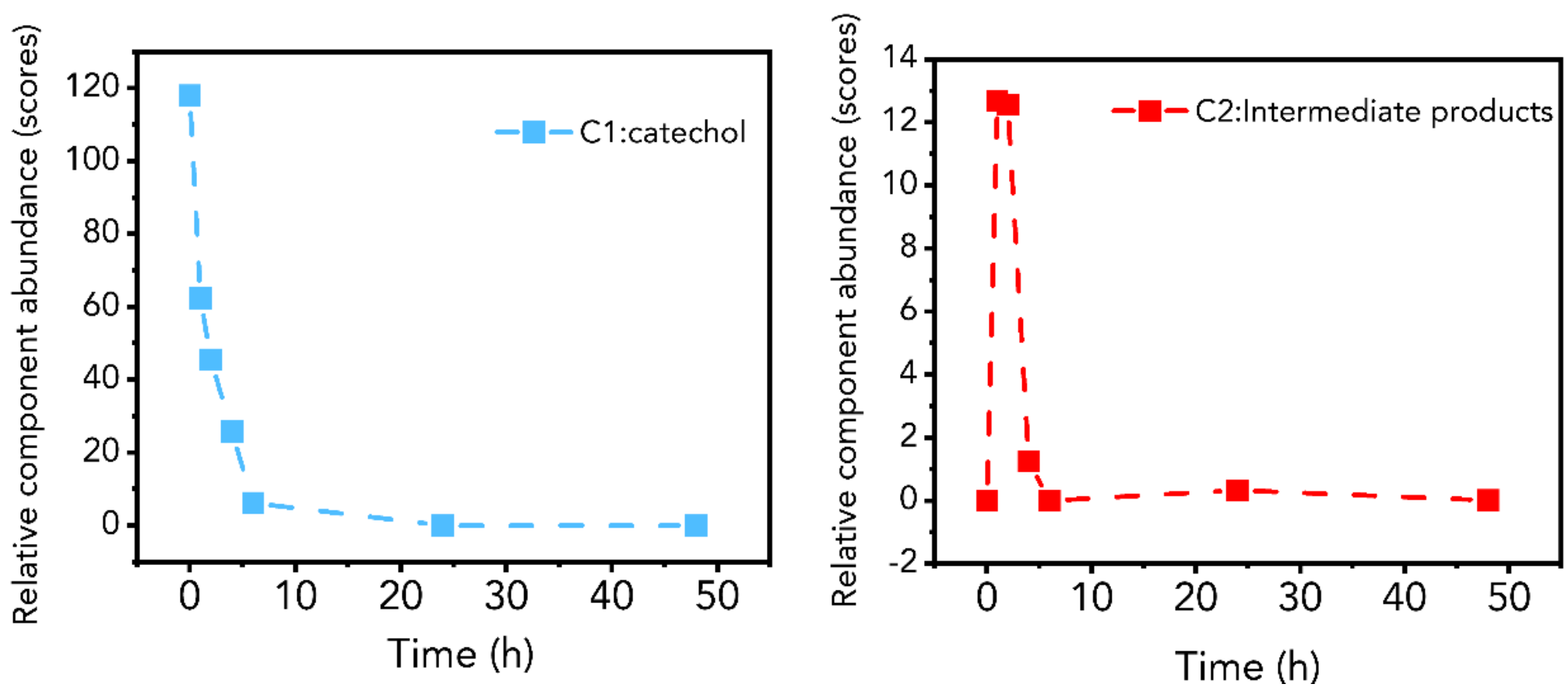


**Figure S22.** Time-dependent variations of components 1 and 2 resolved by PARAFAC. Experimental conditions: $[catechol]_0$ = 50 μM; $[MnO_2]$ = 50 mg $L^{-1}$; $pH_0$ = 7.0, [NaCl] = 1 mM.

**To verify the generalizability of the conclusions in the main text, additional UV-vis and EEM measurements were performed over a range of pH and NaCl conditions, as shown in Figures S23–S29.**

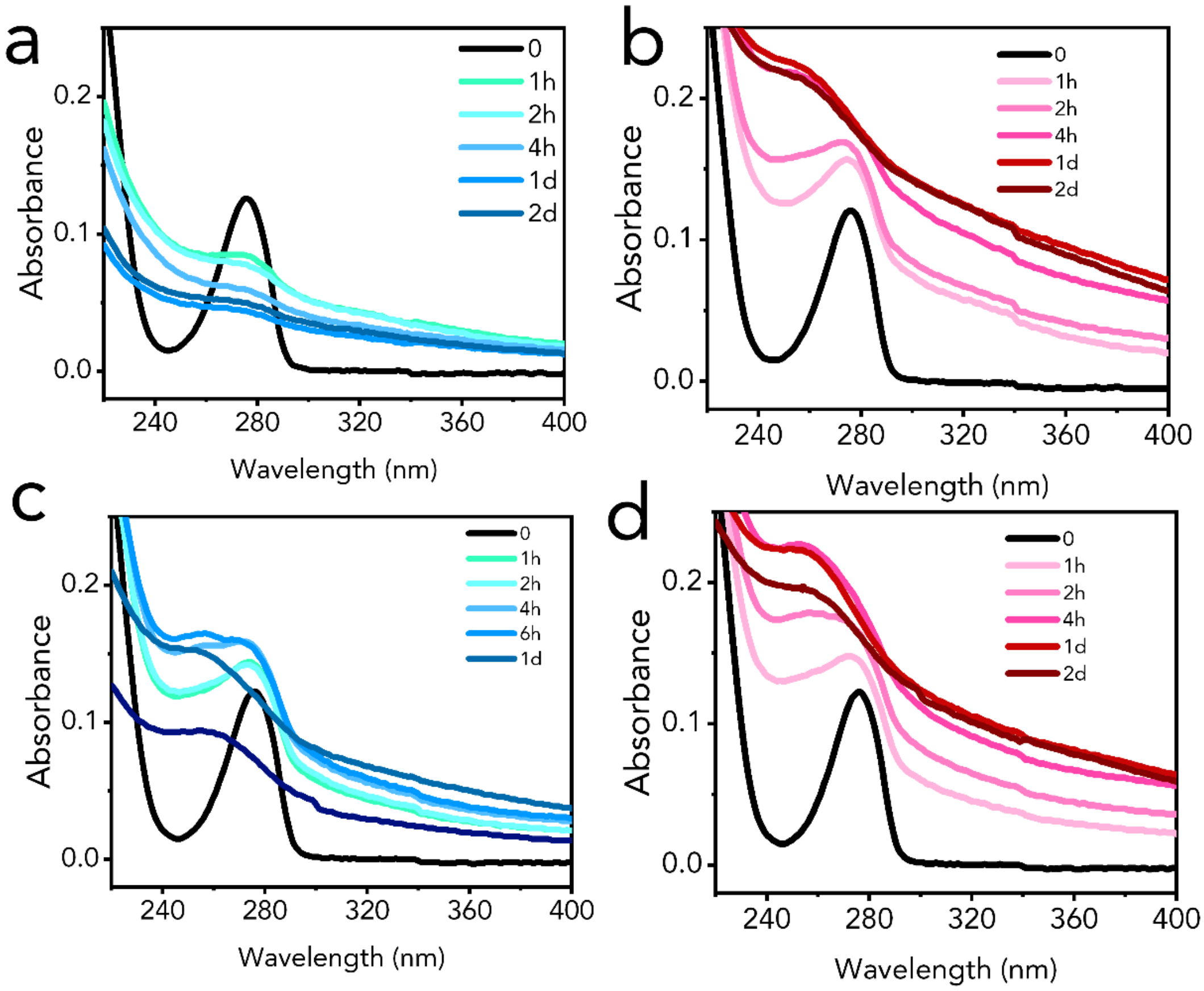


**Figure S23.** Time-resolved UV-vis spectra of the reaction supernatant at 30 mM NaCl under (a) FTCs and (b) RT, and at 100 mM NaCl under (c) FTCs and (d) RT. Experimental conditions: $[\text{catechol}]_0$ = 50 μM; $[MnO_2]$ = 50 mg $L^{-1}$; $pH_0$ = 7.0.

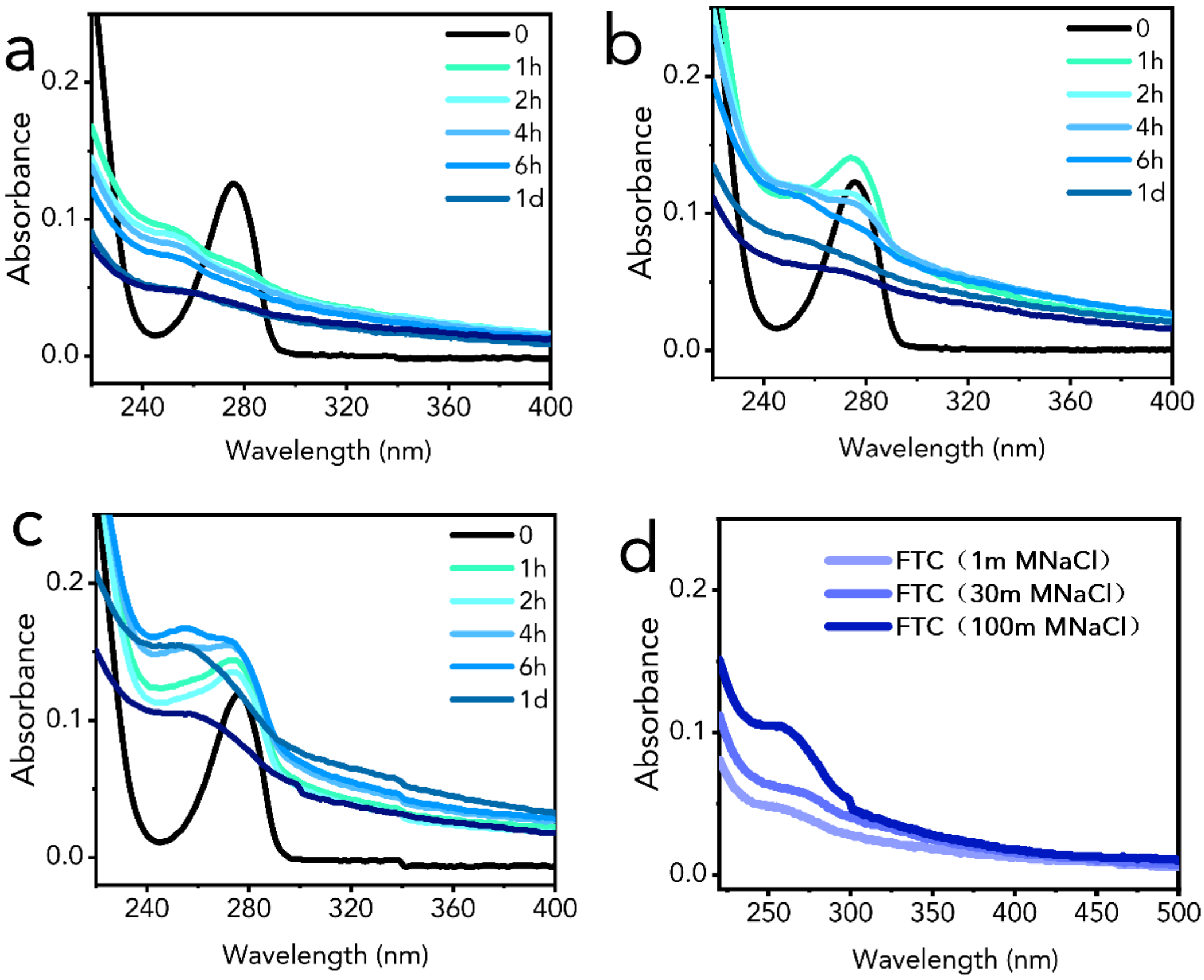


**Figure S24.** Time-resolved UV-vis spectra of the reaction supernatant under FTCs at 1 mM NaCl (a), 30 mM NaCl (b) and 100 mM NaCl (c). (d) UV-vis after two days of reaction under 1–100 mM NaCl. Experimental conditions: $[catechol]_0$ = 50 μM; $[MnO_2]$ = 50 mg $L^{-1}$; $pH_0$ = 5.0.

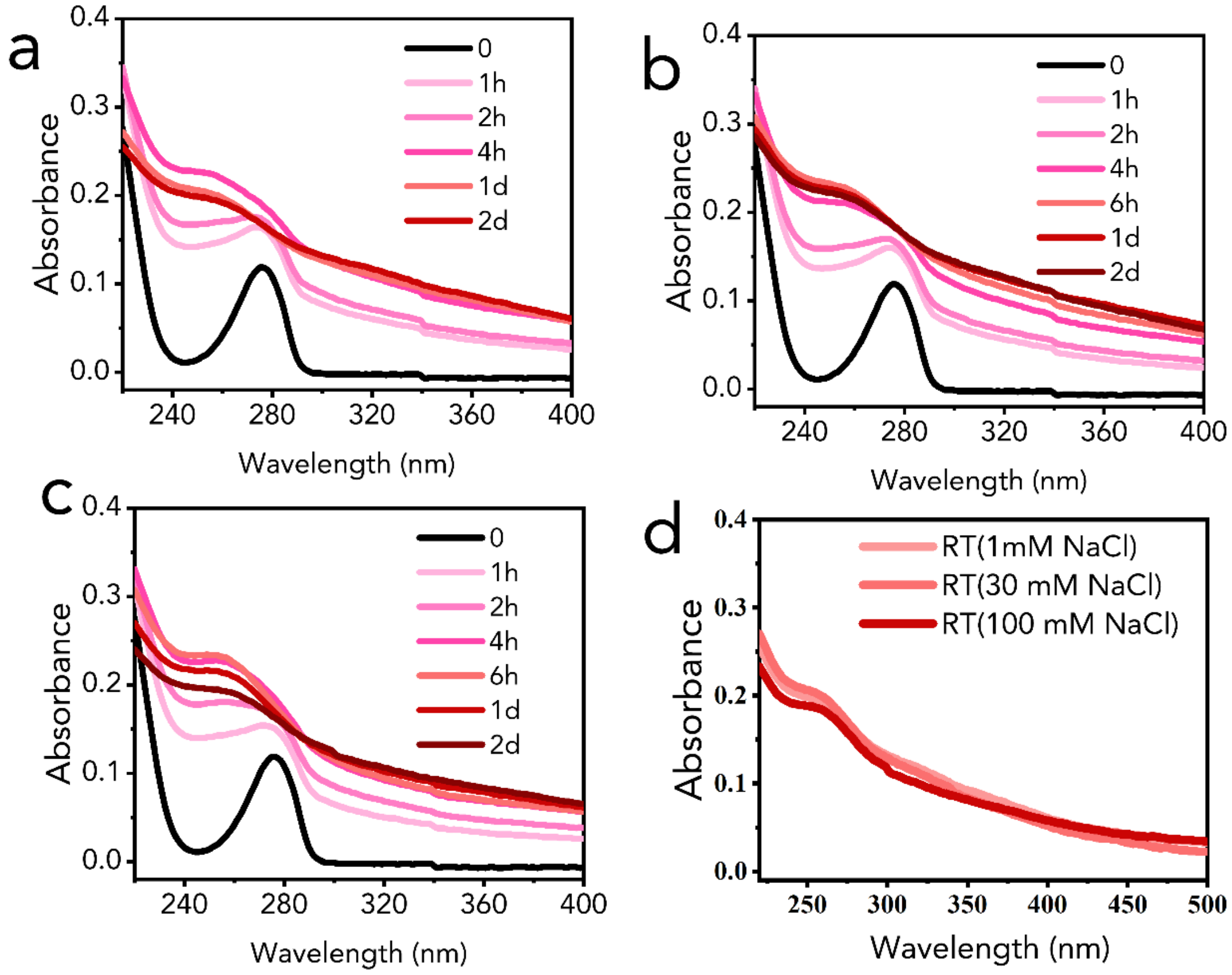


**Figure S25.** Time-resolved UV-vis spectra of the reaction supernatant under RT at 1 mM NaCl (a), 30 mM NaCl (b) and 100 mM NaCl (c). (d) UV-vis after two days of reaction under 1–100 mM NaCl. Experimental conditions: $[catechol]_0$ = 50 μM; $[MnO_2]$ = 50 mg $L^{-1}$; $pH_0$ = 5.0.

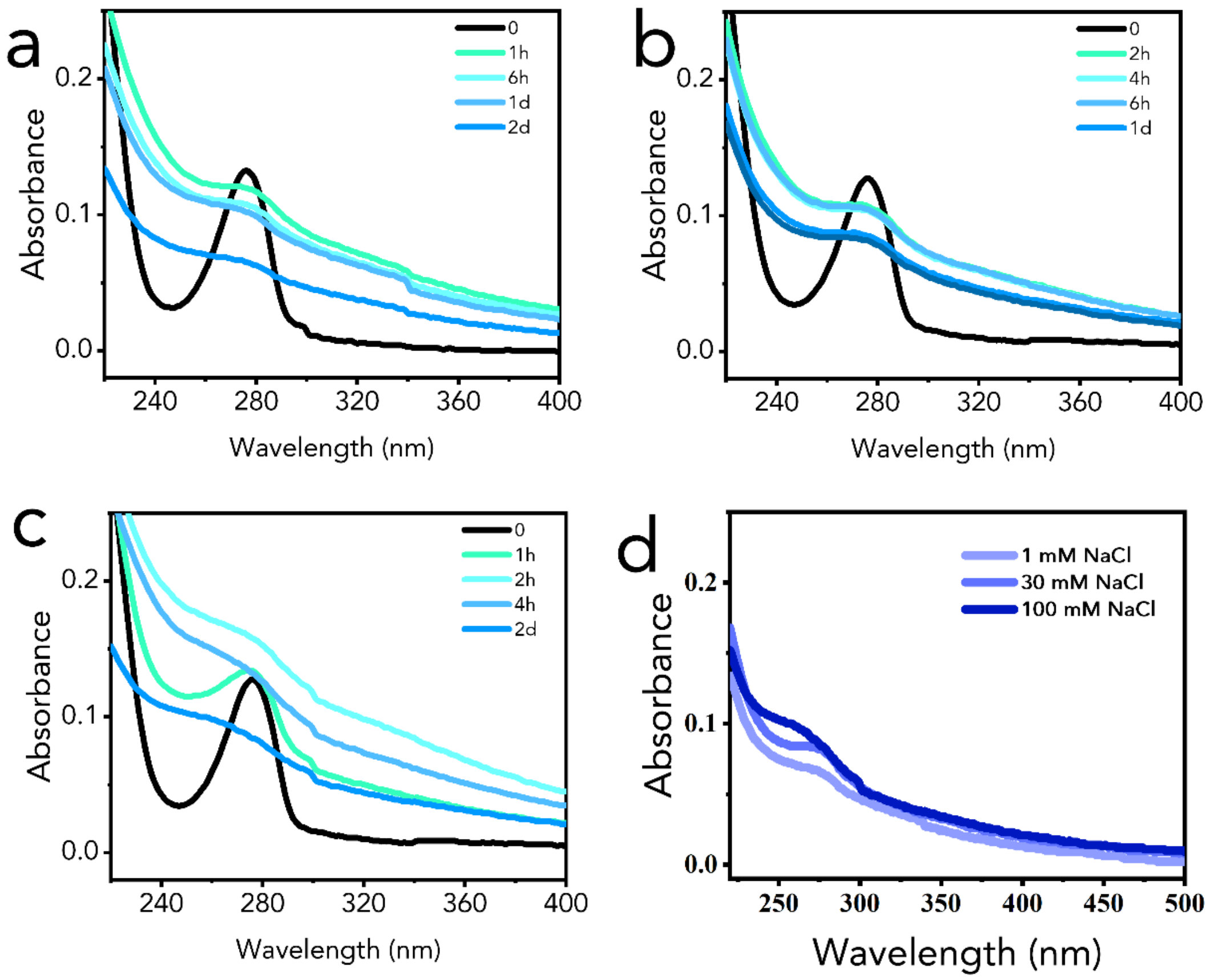


**Figure S26.** Time-resolved UV-vis spectra of the reaction supernatant under FTCs at 1 mM NaCl (a), 30 mM NaCl (b) and 100 mM NaCl (c). (d) UV-vis after two days of reaction under 1–100 mM NaCl. Experimental conditions: $[catechol]_0$ = 50 μM; $[MnO_2]$ = 50 mg $L^{-1}$; $pH_0$ = 9.0.

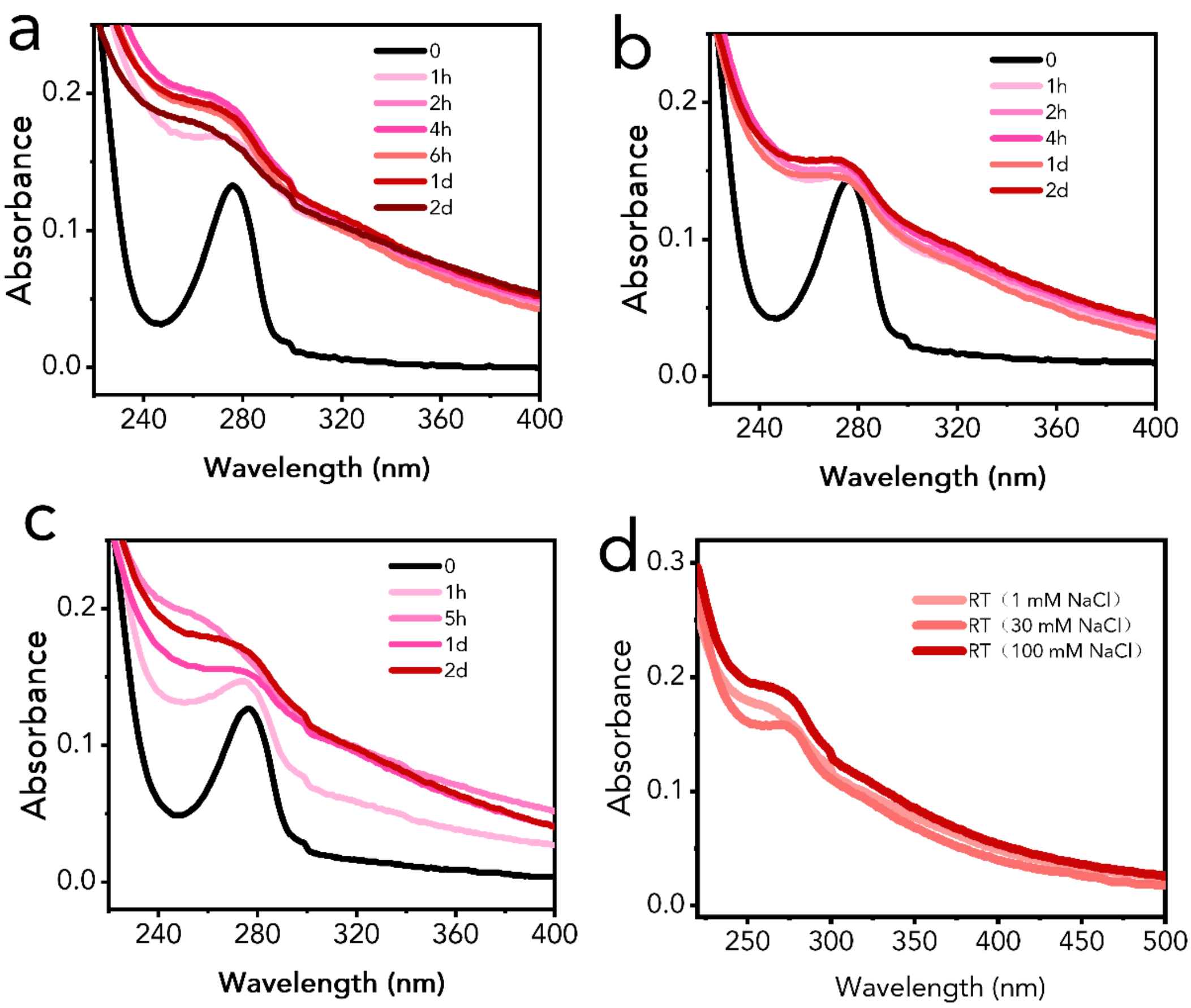


**Figure S27.** Time-resolved UV-vis spectra of the reaction supernatant under RT at 1 mM NaCl (a), 30 mM NaCl (b) and 100 mM NaCl (c). (d) UV-vis after two days of reaction under 1–100 mM NaCl. Experimental conditions: $[catechol]_0$ = 50 μM; $[MnO_2]$ = 50 mg $L^{-1}$; $pH_0$ = 9.0.

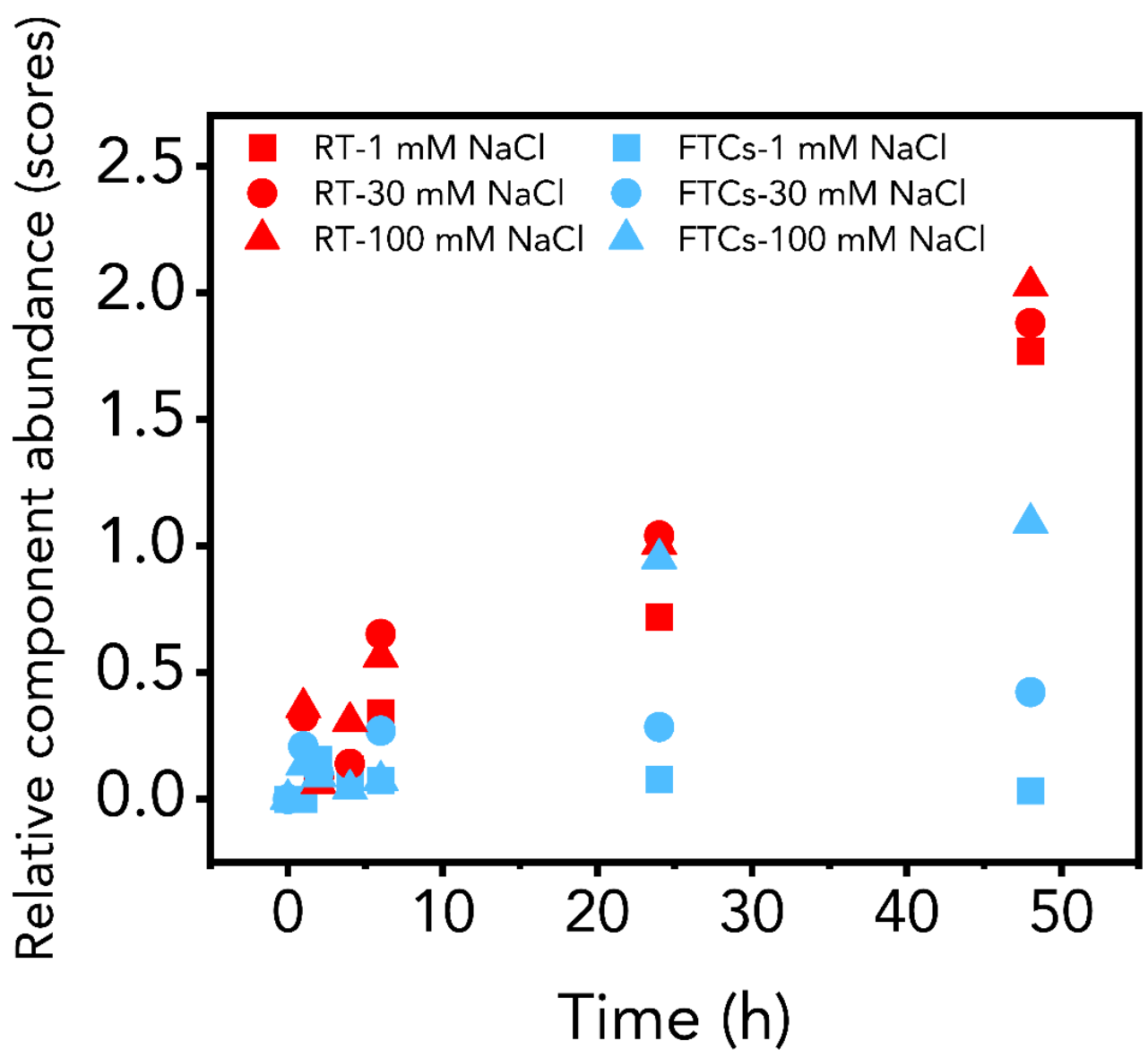


**Figure S28.** Relative component abundance of humic-like acid (HLA) derived from parallel factor analysis (PARAFAC) modeling of the fluorescence spectra. Experimental conditions: $[catechol]_0$ = 50 μM; $[MnO_2]$ = 50 mg $L^{-1}$; $pH_0$ = 5.0.

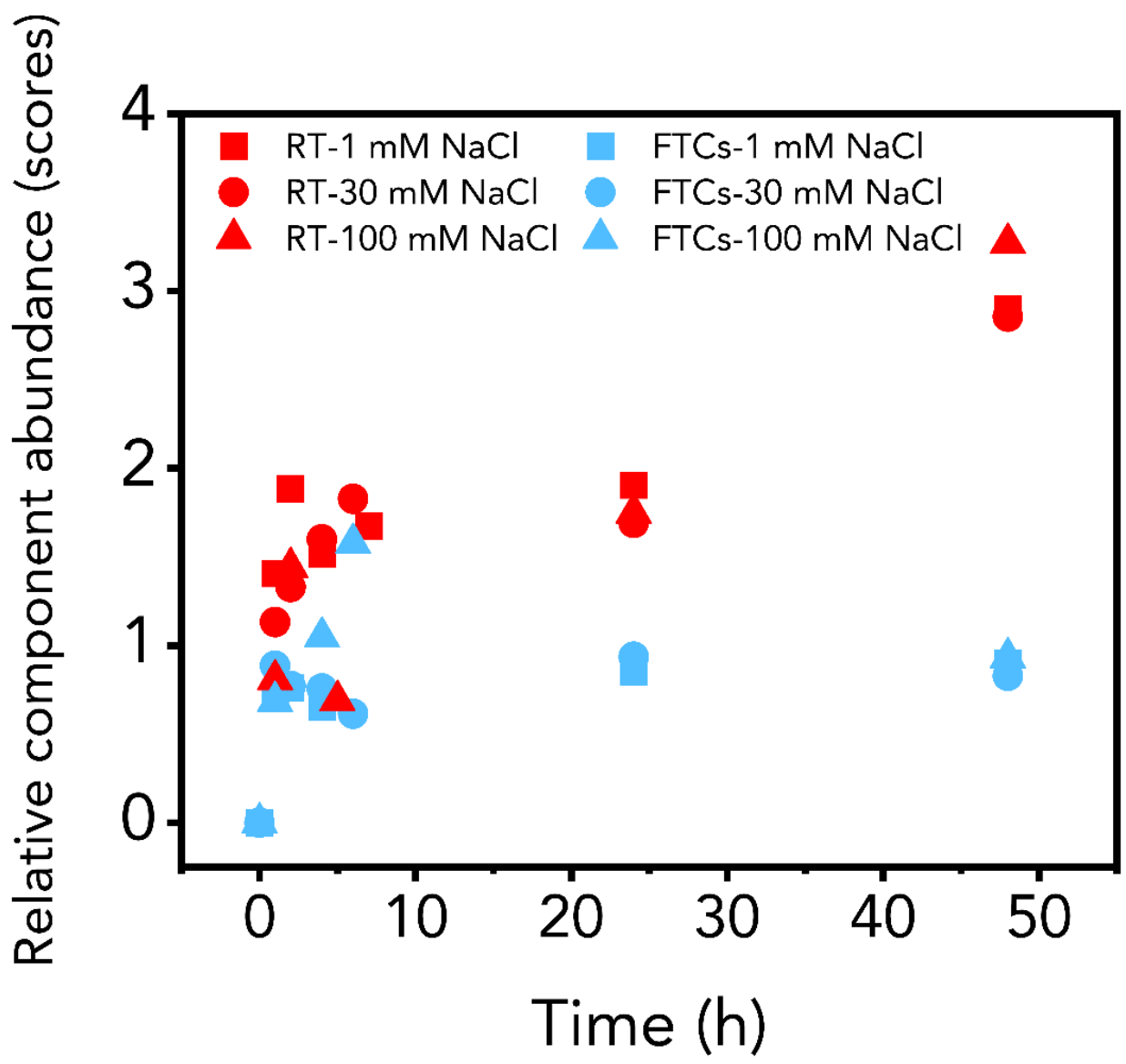


**Figure S29.** Relative component abundance of HLA derived from parallel factor analysis (PARAFAC) modeling of the fluorescence spectra. Experimental conditions: $[catechol]_0$ = 50 μM; $[MnO_2]$ = 50 mg $L^{-1}$; $pH_0$ = 9.0.

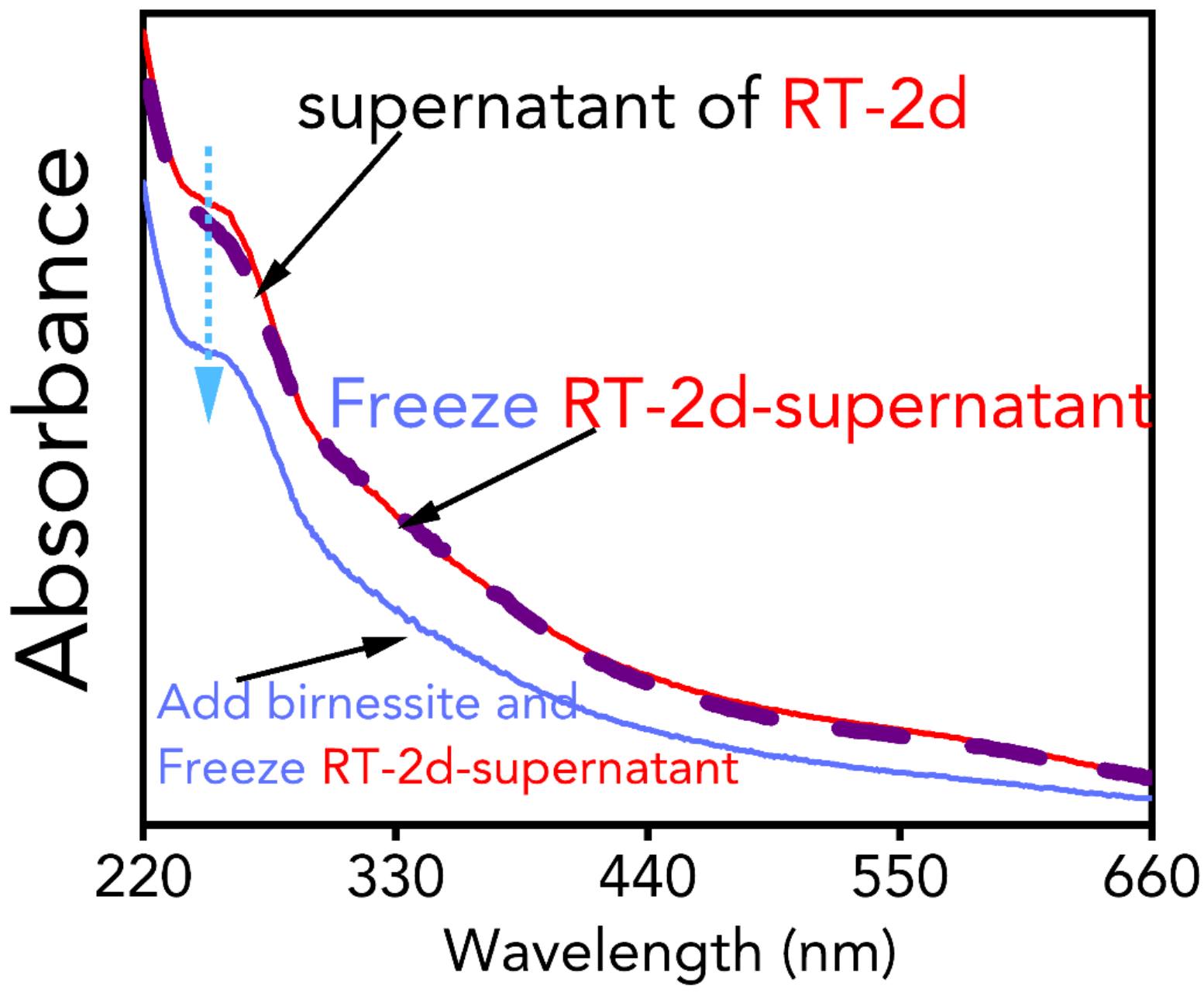


**Figure S30.** UV-vis spectra of the RT supernatant after two days of reaction and after subsequent freeze–thaw treatment with or without birnessite. The red line (RT-2d) represents the UV spectrum of the supernatant after two days of reaction at room temperature. The blue line corresponds to the spectrum obtained by adding 50 mg $L^{-1}$ birnessite to the RT-2d solution followed by one freeze–thaw cycle. The dotted line shows the spectrum obtained by subjecting RT-2d to one freeze–thaw cycle without birnessite addition.

**3. Supplementary Tables**

**Table S1.** XPS spectra and fitting results of Mn $2p_{3/2}$ under FTCs

| | **Position** | **FWHM** | **Raw Area** | **%At Conc** |
|---|---|---|---|---|
| **MnIV-1** | 642.1 | 1.03 | 318.22 | 5.62 |
| **MnIV-2** | 642.88 | 1.03 | 169.93 | 3.00 |
| **MnIV-3** | 643.47 | 1.03 | 157.84 | 2.79 |
| **MnIV-4** | 644.17 | 1.03 | 88.15 | 1.57 |
| **MnIV-5** | 644.95 | 1.03 | 53.14 | 0.95 |
| **MnIV-6** | 645.86 | 1.03 | 27.68 | 0.49 |
| **MnIV-7** | 646.95 | 1.03 | 11.46 | 0.19 |
| **MnIV-8** | 647.17 | 1.03 | 4.14 | 0.08 |
| **MnIII-1** | 641.43 | 1.3 | 919.86 | 16.25 |
| **MnIII-2** | 640.64 | 1.3 | 915.26 | 16.20 |
| **MnIII-3** | 642.11 | 1.3 | 734.05 | 12.98 |
| **MnIII-4** | 642.96 | 1.3 | 568.47 | 10.03 |
| **MnIII-5** | 643.69 | 1.3 | 299.87 | 5.30 |
| **MnIII-6** | 644.53 | 1.3 | 160.98 | 2.84 |
| **MnIII-7** | 645.55 | 1.3 | 67.15 | 1.19 |
| **MnIII-8** | 639.84 | 5.24 | 33.11 | 0.59 |
| **MnII-1** | 640 | 1.3 | 97.8 | 1.73 |
| **MnII-2** | 640.98 | 1.3 | 66.5 | 1.16 |
| **MnII-3** | 641.8 | 1.3 | 49.58 | 0.87 |
| **MnII-4** | 642.68 | 1.3 | 34.82 | 0.62 |
| **MnII-5** | 643.76 | 1.3 | 12.71 | 0.22 |
| **MnII-6** | 644.85 | 1.82 | 9.78 | 0.16 |
| **MnII-7** | 646.05 | 2.75 | 858.04 | 15.12 |
| **MnII-8** | 638.35 | 1.3 | 3.81 | 0.05 |

**Table S2.** XPS spectra and fitting results of Mn $2p_{3/2}$ under RT

| | Position | FWHM | Raw Area | %At Conc |
|---|---|---|---|---|
| **MnIV-1** | 641.93 | 1.3 | 4397.19 | 18.74 |
| **MnIV-2** | 642.71 | 1.3 | 2348.1 | 10.01 |
| **MnIV-3** | 643.3 | 1.3 | 2181.01 | 9.28 |
| **MnIV-4** | 644 | 1.3 | 1218.02 | 5.19 |
| **MnIV-5** | 644.78 | 1.3 | 734.33 | 3.13 |
| **MnIV-6** | 645.69 | 1.3 | 382.56 | 1.62 |
| **MnIV-7** | 646.78 | 1.3 | 158.3 | 0.67 |
| **MnIV-8** | 647 | 1.3 | 57.16 | 0.24 |
| **MnIII-1** | 641.53 | 1.1 | 1970.48 | 8.40 |
| **MnIII-2** | 640.74 | 1.1 | 1960.63 | 8.37 |
| **MnIII-3** | 642.21 | 1.1 | 1572.44 | 6.70 |
| **MnIII-4** | 640.68 | 1.1 | 1217.76 | 5.19 |
| **MnIII-5** | 643.79 | 1.1 | 642.38 | 2.73 |
| **MnIII-6** | 644.63 | 1.1 | 344.83 | 1.46 |
| **MnIII-7** | 645.65 | 1.1 | 143.85 | 0.61 |
| **MnIII-8** | 639.94 | 4.58 | 70.94 | 0.31 |
| **MnII-1** | 640 | 1.3 | 1134.78 | 4.85 |
| **MnII-2** | 640.98 | 1.3 | 771.65 | 3.29 |
| **MnII-3** | 641.8 | 1.3 | 575.33 | 2.46 |
| **MnII-4** | 642.68 | 1.3 | 403.98 | 1.72 |
| **MnII-5** | 643.76 | 1.3 | 147.52 | 0.63 |
| **MnII-6** | 644.85 | 1.82 | 113.48 | 0.48 |
| **MnII-7** | 646.05 | 2.75 | 874.95 | 3.73 |
| **MnII-8** | 638.35 | 1.3 | 44.26 | 0.19 |

**Table S3.** XPS fitting results and Mn-normalized values for pure birnessite, FTC, and RT solids.

| | | **Pure birnessite** | **FTC** | **RT** |
|---|---|---|---|---|
| **C** | C-(C,H) **BE scale reference** | 7.2 | 18.0 | 10.9 |
| | C-OH | 1.6 | 6.3 | 4.0 |
| | COOH | 2.2 | 3.9 | 3.1 |
| **O** | C=O, Mn-OH | 3.69 | 15.07 | 15.44 |
| | C-OH | 0 | 14.10 | 8.56 |
| **Mn** | Mn 2p 3/2 | 33.1 | 23.2 | 28.8 |
| **C/Mn** | C-(C,H) **BE scale reference** | 0.218 | 0.776 | 0.378 |
| | C-OH | 0.048 | 0.272 | 0.139 |
| | COOH | 0.066 | 0.168 | 0.108 |
| **O/Mn** | C=O, Mn-OH | 0.111 | 0.650 | 0.536 |
| | C-OH | 0 | 0.608 | 0.297 |

**Table S4.** Mn oxidation-state distributions for pure birnessite, FTC, and RT solids determined by XPS.

| | **Pure birnessite** | **FTC** | **RT** |
|---|---|---|---|
| | | | |
| **Mn(II) (at.%)** | 4.0 | 20.0 | 17.3 |
| **Mn(III) (at.%)** | 22.6 | 65.4 | 33.8 |
| **Mn(IV) (at.%)** | 73.4 | 14.7 | 48.9 |

**4. References**


1. K. R. Murphy, C. A. Stedmon, D. Graeber, R. Bro, Fluorescence spectroscopy and multi-way techniques. PARAFAC. *Analytical Methods* **5**, 6557–6566 (2013).
2. E. S. Ilton, J. E. Post, P. J. Heaney, F. T. Ling, S. N. Kerisit, XPS determination of Mn oxidation states in Mn (hydr)oxides. *Appl. Surf. Sci.* **366**, 475–485 (2016).
3. J. Bijlsma, *et al.*, Revealing the main factors and two-way interactions contributing to food discolouration caused by iron-catechol complexation. *Sci. Rep.* **10**, 8288 (2020).
4. L. Curti, *et al.*, Carboxyl-richness controls organic carbon preservation during coprecipitation with iron (oxyhydr)oxides in the natural environment. *Commun. Earth Environ.* **2**, 229 (2021).
5. A. Siregar, M. Kleber, R. Mikutta, R. Jahn, Sodium hypochlorite oxidation reduces soil organic matter concentrations without affecting inorganic soil constituents. *Eur. J. Soil Sci.* **56**, 481–490 (2005).
